# A Comprehensive Review of a Mechanism-Based Ventricular Electrical Storm Management

**DOI:** 10.3390/jcm14155351

**Published:** 2025-07-29

**Authors:** Alina Gabriela Negru, Diana Carina Iovanovici, Ana Lascu, Alexandru Silviu Pescariu, Gabriel Cismaru, Simina Crișan, Ștefan Ailoaei, Diana Luiza Bebec, Caius Glad Streian, Mariela Romina Bîrza, Andrei Raul Manzur, Silvia Ana Luca, Dana David, Svetlana Moșteoru, Dan Gaiță, Constantin Tudor Luca

**Affiliations:** 1 Cardiology II Department, Victor Babes University of Medicine and Pharmacy, 300041 Timișoara, Romania; pescariu.alexandru@umft.ro (A.S.P.); simina.crisan@umft.ro (S.C.); silvia.luca@umft.ro (S.A.L.); dgaita@cardiologie.ro (D.G.); ctluca@cardiologie.ro (C.T.L.); 2 Rocordis Heart Center, 300278 Timișoara, Romania; dia.luiz19@gmail.com (D.L.B.); romina.birza@umft.ro (M.R.B.); 3 Advanced Research Centre of the Institute of Cardiovascular Diseases, Victor Babes University of Medicine and Pharmacy, 300310 Timișoara, Romania; lascu.ana@umft.ro (A.L.); tanaly@gmail.com (S.M.); 4 Institute of Cardiovascular Diseases Timișoara, Clinic of Cardiology, 300310 Timișoara, Romania; 5 Doctoral School of Biomedical Sciences, University of Oradea, 410087 Oradea, Romania; 6 Department III of Functional Sciences, Discipline of Pathophysiology, Victor Babes University of Medicine and Pharmacy, 300041 Timișoara, Romania; 7 Department VI Cardiovascular Surgery, Victor Babes University of Medicine and Pharmacy, 300041 Timișoara, Romania; streian.caius@umft.ro; 8 Institute of Cardiovascular Diseases Timișoara, Clinic of Cardiovascular Surgery, Victor Babes University of Medicine and Pharmacy, 300310 Timișoara, Romania; 9 Department of Cardiac Rehabilitation Cluj, University of Medicine and Pharmacy “Iuliu Hațieganu”, 400012 Cluj-Napoca, Romania; gabi_cismaru@yahoo.com; 10 Cardiology Department, University of Medicine and Pharmacy “Gr. T. Popa”, 700115 Iași, Romania; stefan.ailoaei@yahoo.com; 11 Institute of Cardiovascular Diseases “Prof Dr. George I.M Georgescu”, 700503 Iași, Romania; 12 Center for Research and Innovation in Precision Medicine of Respiratory Diseases, Department of Pulmonology, Victor Babes University of Medicine and Pharmacy, 300041 Timișoara, Romania; andrei.manzur@umft.ro; 13 Doctoral School Medicine-Pharmacy, Victor Babes University of Medicine and Pharmacy, 300041 Timișoara, Romania; 14 Department of Biochemistry, Victor Babes University of Medicine and Pharmacy, 300041 Timișoara, Romania; david.dana@umft.ro

**Keywords:** electrical storm, sudden death, ventricular arrhythmia

## Abstract

The electrical ventricular storm (VES) is defined as multiple sustained ventricular arrhythmias arising in a short time, often refractory to standard antiarrhythmic treatment. The three pillars of the physiopathogenesis of the VES are autonomic dysfunction, triggers, and an altered ventricular substrate. Incessant or highly recurrent ventricular arrhythmia impacts the hemodynamic status by worsening heart failure and increasing mortality. A stepwise, team-based, and tailored therapeutic approach is required to stop ventricular arrhythmia and regain the hemodynamic and electric stability of the patient. The authors focused on describing all currently available therapeutic approaches for VES, intending to establish the best VES therapeutic approaches. This process involves considering the patient’s specific condition, responses to previous treatments, and the potential risks and benefits of each approach. The options range from adjusting antiarrhythmic therapy to reprogramming of the ICD, sedation, epidural anaesthesia, stellate ganglia anaesthetic block, and the use of ECMO or left ventricular assist devices and radiofrequency catheter ablation. Particular attention is paid to the detailed management of genetic primary arrhythmia syndromes like long-QT syndrome, catecholaminergic polymorphic ventricular tachycardia, Brugada syndrome and Wolff–Parkinson–White syndrome, early repolarisation syndrome, right ventricular arrhythmogenic dysplasia, and idiopathic ventricular fibrillation. After overcoming the acute events of VES and obtaining hemodynamic stability, the treatment should shift toward an optimal balance of heart failure therapy, controlling the substrate by revascularisation procedures and resolving other pathology-generating ventricular arrhythmias. This article provides a comprehensive overview of ESV’s current management options using the most efficient strategies known to date.

## 1. Introduction

A ventricular electrical storm (VES) is a state of cardiac electrical instability associated with a high risk of death during the episode or the follow-up, ranging from 13% to 55% over 2.75 to 5 years [1]. There is a consensus regarding the definition of an electrical storm, which is referred to as incessant, life-threatening ventricular arrhythmia or three or more ventricular arrhythmia episodes occurring more than 5 min apart within 24 h, associated with hemodynamic instability, and requiring intervention for termination [2,3]. Even if the association between VES and increased mortality is well known, a recent study demonstrated highly significant mortality rates also associated with clustered ventricular arrhythmia, starting from 2 episodes within three months in patients with implantable cardioverter defibrillators (ICDs), and increasing proportionally with the number of arrhythmic episodes [4]. In patients wearing an ICD, VES represents a major medical emergency as well, presenting with repeated anti-tachycardia pacing and/or multiple internal electrical shocks. The ventricular arrhythmias causing ventricular storms are most frequently sustained monomorphic or polymorphic ventricular tachycardia, bidirectional ventricular tachycardia, torsade de pointes (TdP), and ventricular fibrillation. However, over 80% of all VESs are caused by monomorphic ventricular tachycardia. The risk of mortality in patients with VES is 2.5-fold greater than in patients with non-clustered, isolated ventricular tachycardias and 3.3-fold more significant than in patients with non-sustained ventricular arrhythmias during a similar follow-up period [5]. Moreover, a mortality rate of up to 14% was described in the first 48 h after the onset of VES and a 5.6-fold increase in mortality during the first 12 weeks following VESs in patients with ICDs implanted for secondary prevention [6,7].

### 1.1. Historical and Mechanistic Perspectives of Ventricular Electrical Storm

The concept of VES has evolved significantly since its initial recognition as a group of life-threatening ventricular arrhythmias. Early reports in the 1990s described VES primarily in patients with ICDs, where recurrent shocks revealed a pattern of electrical instability. Over time, the definition has been refined to include ≥3 episodes of sustained VT or VF within 24 h that require intervention. Initially considered a rare phenomenon, VES is now recognised as a distinct clinical entity with serious prognostic implications, particularly in patients with structural heart disease or inherited channelopathies [8,9].

Preclinical models have helped elucidate the pathophysiology of VES, with animal studies showing that sympathetic overactivation, aberrant calcium manipulation, and re-entry circuits in myocardial scar tissue are essential for the development and persistence of arrhythmias. Experimental models using ischemia–reperfusion lesions, genetic manipulation of ion channels, and autonomous modulation have revealed key molecular factors such as CaMKII activation, early postdepolarisations, and modified repolarization gradients [10,11,12].

### 1.2. Epidemiology

VES is a critical clinical challenge in patients with ICDs, with its incidence varying according to population characteristics and follow-up duration. There is a notably higher prevalence among patients receiving ICDs for the secondary prevention compared to the primary prevention of sudden cardiac death. For instance, in patients with an ICD for secondary prevention of sudden cardiac death, the incidence is between 10 and 30% whereas for patients with an ICD in primary prevention, it is between 4 and 7%, evaluated 18 to 24 months after device implantation [8,13]. In a separate study, 20% of patients with ICDs implanted for secondary prevention developed electrical storms over a follow-up duration of 31 months [14]. The arrhythmic burden typically emerges within 6 to 36 months following ICD implantation and is most frequently triggered by monomorphic ventricular tachycardia, although ventricular fibrillation (VF) may also occur. Most VES episodes occur in patients with underlying structural heart disease. This is most commonly ischemic heart disease (77–94%) but also occurs in patients with primary electrical disturbances [3,15].

Consistent data on the incidence of VES in individuals with no implanted cardiac devices are limited and highly dependent on the presence or absence of cardiovascular substrates, associated triggers, and the specific population being studied. One longitudinal study reported a VES incidence of 4.7% over a median follow-up of 39 months, while a meta-analysis found that 13% of VES patients experience recurrent episodes [16,17].

VES is most commonly associated with structural heart disease, particularly ischemic and non-ischemic cardiomyopathies, with comparable event rates in both groups [18]. Although rare, VES has also been documented in individuals without overt structural abnormalities [19].

Prognostically, VES portends an unfavourable trajectory, conferring elevated risks of all-cause mortality, heart failure hospitalisation, major adverse cardiac events, and significantly poorer survival compared to patients with isolated or no ventricular arrhythmias. VES may act as an independent predictor of SCD. Moreover, its presence in the context of acute myocardial infarction is associated with worsened clinical outcomes. Comorbidities such as chronic kidney disease, reduced LVEF, and the use of amiodarone further compound the adverse prognostic impact of VES [16,20,21]. Over time, various authors have identified cardiac and extracardiac risk factors for VES, with the most common being a low LVEF, a prolonged QRS duration, and a history of ventricular arrhythmia episodes (Table 1).

In the case of non-ischemic cardiomyopathy, although less frequent, VES remains clinically relevant. In a cohort of arrhythmogenic cardiomyopathy (ACM) patients with ICDs, the incidence reached 21.6%, with predictors including high BMI and extensive T-wave inversion (Table 1).

The early post-MI period is associated with heightened arrhythmic vulnerability due to scar-related re-entry circuits. VES may occur in up to 10% of high-risk post-MI patients, especially those with residual ischemia or poor ventricular remodelling.

Regarding patients with channelopathies, inherited electrical disorders such as Brugada syndrome, long QT syndrome, and catecholaminergic polymorphic VT contribute disproportionately to VES in younger populations. These conditions may present without structural heart disease but carry a high risk of SCD.

## 2. Methods

This narrative review was conducted with a structured approach to ensure comprehensive and relevant coverage of the literature on VES management. A literature search was performed using PubMed, EMBASE, and Google Scholar for articles published between January 1990 and March 2025. The following search terms and Boolean operators were employed: “ventricular electrical storm” or “electrical storm” and (“mechanism-based management” or “catheter ablation” or “sympathetic modulation” or “antiarrhythmic therapy”) and (“molecular mechanisms electrical storm” or “primary genetic arrhythmic syndromes”).

Inclusion Criteria:

- Peer-reviewed articles, clinical trials, meta-analyses, and authoritative reviews.
- Studies discussing pathophysiological mechanisms, diagnostic considerations, or interventional strategies specifically targeting VES.
- Publications in English.

Exclusion Criteria:

- Case reports with fewer than three patients.
- Abstracts without full-text availability.
- Non-human studies, with the exception of those directly relevant to mechanisms or interventions later validated in clinical settings.

To maintain the narrative nature of the review, the selection and synthesis of sources were guided by their thematic relevance and the potential to contribute mechanistic insights or recommendations of practical management strategies for clinicians.

## 3. Physiopathogenesis of the Electrical Ventricular Storm

The conceptual framework known as “Coumel’s triangle of arrhythmogenesis” continues to be the primary model illustrating how the dynamic interaction among trigger, substrate, and modulator contributes to the development of arrhythmia [24]. Understanding the mechanisms underlying VES is key to optimal treatment and the most rapid possible recovery, considering that the degree of involvement and the diversity of mechanisms may vary significantly in producing malignant ventricular arrhythmias at a given time. The three pillars of the physiopathogenesis of the VES are triggered activity, autonomic dysfunction, and altered ventricular substrate [25,26].

*Triggered activity*: Understanding normal cardiac cellular electrophysiology is the basis for recognising abnormally triggered activity. The normal action potential consists of five phases (Table 2) interplayed by three primary ions: the sodium (Na^+^), potassium (K^+^) and calcium (Ca^2+^) ions. Cardiac myocytes are capable of myocardial contraction, automaticity, and electrical conduction of the impulses [27]. Spontaneously generated action potentials are propagated through excitable cardiomyocytes. Excitability is the capacity of the cell to respond to electrical stimuli provoked by a regenerative action potential [28].

A combination of prolonged action potential and early or delayed after-depolarisations (ADs) is mandatory for the triggered activity. The ADs are abnormal depolarisations determined in the excito-conductory system, interrupting phases 2, 3, or 4 of the action potential. Early ADs usually originate in the Purkinje fibres or myocytes and occur during phases 2 and 3 of the action potential, having the potential to trigger new action potentials. The pathological implication of early ADs lies in their double impact on the ventricular myocardium as a trigger (ventricular ectopic beats) and as a destabiliser of the myocardial electricity (causing electrical heterogeneity). The late ADs begin when phase 4 of the action potential is almost or entirely accomplished, but before the beginning of the following action potential.

*Autonomic dysfunction*: The autonomic nervous system plays a crucial role in the heart’s adaptive functions, such as inotropy, chronotropy and dromotropy [29]. Autonomic imbalance usually acts as sympathetic tone stimulation or adrenergic drive and parasympathetic abolition when providing the environment for electrical instability and ventricular arrhythmia initiation. The cardiac neuraxis is a complex structure that receives, integrates and adapts the afferent inputs to continuously generate a double sympathetic and parasympathetic response for the appropriately adapted heart function. The cardiac neuraxis consists of the intrinsic cardiac nervous system, intrathoracic extracardiac components and the central nervous system. The intrinsic cardiac nervous system consists of several ganglionated plexi situated on the external part of the myocardium at the level of epicardial fat pads [30]. The intrathoracic extracardiac nervous system consists of intrathoracic ganglia (stellate and cervical ganglia) with their parasympathetic and sympathetic motor neurons, providing the link between the cardiac and central nervous systems. [31]. During sympathoexcitation, epinephrine and norepinephrine secreted by sympathetic neurons bind to the beta-receptors, activating at first cAMP and then protein kinase A. The protein kinase A phosphorylates the L-type calcium channel, resulting in a subsequent calcium influx. At the same time, calcium released from the sarcoplasmic reticulum is increasing due to a greater calcium loading, resulting in enhanced myocardial contractility and a higher incidence of early AD or delayed AD. Basically, the ADs are favoured by the L-type calcium channel reactivation during a prolonged phase 2 of the action potential or by the sodium-calcium exchanger, an antiporter protein located in the membrane that removes calcium from the cell. Moreover, early AD and the prolongation of action potential duration are more likely to occur because the subsequent sodium current and slow potassium current activation cannot efficiently counterbalance the effect of calcium overload at the beginning of the activation [32]. Re-entry can also be an effect of sympathoexcitation due to the shortening of the effective refractory period caused by slow potassium channel activation, simultaneous with the electrical repolarisation dispersion, especially in the hyperinervated regions.

Altered ventricular substrate and autonomic imbalance can be responsible for enhanced or abnormal automaticity and triggered activity leading to VES. The abnormal automaticity of ventricular non-pacemaker cells is influenced by the interaction of the value of maximal diastolic potential driven over the threshold potential capable of AP initiation and the duration of phase 4 of the depolarisation slope [33].

### 3.1. The Triggers

#### 3.1.1. Electrolyte Imbalance

Electrolyte imbalances such as hypomagnesemia, hypokalemia, hypocalcemia, as well as their opposites—hyperkalemia, hypermagnesemia, and hypercalcemia—can lead to ventricular arrhythmias, especially in the presence of substrate alteration or primary arrhythmogenic conditions. In the absence of dyselectrolytemias correction, the cessation of VES becomes more challenging to achieve. Hypomagnesemia, hypokalemia, and even hypocalcemia can be linked to TdP due to their association with prolongation of the QT interval and widespread T wave dispersion [13].

#### 3.1.2. Inflammation, Infection and Fever

Various mechanisms associated with COVID-19 and other viral infection-related VES have been identified. The primary mechanisms appear to involve inflammation and injury to cardiac muscle, evidenced by elevated cardiac markers (Tr I HS, Tr T), in the setting of myocarditis, which can be caused either directly by the virus’s cytolytic effects or indirectly through a hyperinflammatory response associated with a cytokine storm. Other incriminating mechanisms implicated in VES genesis in patients with SARS-CoV-2 pneumonia are hypoxemia, acidosis and a systemic hyperadrenergic response [34].

#### 3.1.3. Hypoxia, Hypercapnia and Acidosis

Hypoxia, hypercapnia, and acidosis are linked to pulmonary pathologies such as chronic obstructive lung disease, pneumonia, and pulmonary embolism. Sinus tachycardia is a response to hypoxia, managing to compensate for the imbalance up to a certain point and increasing oxygen demand. Hypoxia induces the malfunction of all ion channels implicated in action potential generation, as the L-type calcium channel causes a decrease in the basal current carried by this channel and also sodium channels [35].

The decrease in the partial pressure of oxygen (PaO2) increases the activity of Na_v_ 1.5, the primary cardiac voltage-gated Na^+^ channel, which will enhance the late sodium current I (Na-L), leading to an increase in the action potential duration (APD). The prolonged APD creates the substrate for early after-depolarisations (EADs) by allowing L-type calcium channels to recover their activation. At the same time, the cellular membrane is still depolarised during a prolonged phase 2 [36]. On the other hand, a prolonged APD increases the transmural dispersion of depolarisation, leading to refractory obstacles, unidirectional block, and eventually re-entry.

The effect of decreased PaO2 and acidosis is a reduction in the production of ATP, which results in the lowering of the ATP-sensitive potassium channel (IK_ATP_) responsible as well for the prolongation of ADP, EADs, and re-entry [37]. Hypoxia may affect ion channel functioning through changes in reactive oxygen species (ROS), which include oxygen-derived molecules such as superoxides, hydroxyl radicals, ozone and hydrogen peroxide [35,38]. The complex interplay between ionic currents, membrane potentials, and cellular processes is relevant for the induction of VES.

#### 3.1.4. Alteration of SUMOylation

Another essential aspect of the influence of stressors on cardiac cell function is the alteration of the SUMOylation-mediated proteolytic mechanism. Misfolded or damaged proteins generated excessively as a response to cardiovascular stress are cleared by the SUMOylation-mediated mechanism, ubiquitin-proteosome system and autophagy. SUMOylation is also responsible for modulating the ion channels and influencing mitochondrial dynamics. This means that once the proteolytic mechanisms are damaged, unfolded mechanisms accumulate in the cardiomyocyte, leading to cell dysfunction [39]. The SUMO protein group can be implicated in the genesis of ventricular arrhythmias by influencing the ion channel function. The IKs channels, slowly activating potassium currents, play a vital role in ventricular repolarisation [40]. A recent study highlights an intriguing finding: all four KCNQ1 subunits of the IKs channel can potentially undergo SUMOylation. Interestingly, each SUMO molecule added shifts the half-maximum activation voltage toward the positive side. This dynamic modification could have significant implications for cardiac electrophysiology [41]. Kv1.5 channels play a crucial role in cardiomyocytes by mediating the ultra-rapid delayed rectifier potassium current. This current contributes to the repolarisation phase of the cardiac action potential, ensuring proper electrical stability and rhythm. The SUMOylation of these channels finally controls ventricular excitability [42]. SUMOylation is also crucial as the “gatekeeper” for K2P1 channels. These channels are essential for maintaining the resting membrane potential of cells. By modulating K2P1 channels, SUMOylation contributes to cellular excitability and overall membrane stability and the reverse in the case of SUMO alteration [43].

#### 3.1.5. Adrenergic Drive

Increased sympathetic tone is associated with the release of catecholamines binding to beta-receptors in myocytes, leading to a succession of processes responsible for increased conduction velocity, contractility and heart rate [31]. Events like acute ischemia, heart failure decompensation, cardiac arrest and even ICD shocks themselves can increase sympathetic tone. Adrenergic activation plays a critical role in influencing ventricular arrhythmias by altering the electrophysiological and intracellular dynamics of myocardial cells. Its impact can be understood through three primary mechanisms: increased electrical excitability, changes in electrical conduction, and disruptions in calcium handling.

Catecholamines like epinephrine and norepinephrine bind to beta-adrenergic receptors on cardiac cells, triggering a cascade that enhances ion channel activity for depolarisation, such as Na+ and Ca^2+^ channels. This shortens the refractory period. As it decreases, myocardial cells become more excitable and susceptible to premature beats, triggering sustained arrhythmias, especially in patients with fibrosis or scarring. Adrenergic activation alters electrical impulse propagation in the myocardium by affecting conduction velocity, increasing speed in some areas while slowing it in others, creating conduction heterogeneity. These discrepancies facilitate the development of re-entrant circuits, which are crucial for initiating and perpetuating sustained ventricular arrhythmias [44,45]. 

Adrenergic stimulation significantly affects intracellular calcium handling via cyclic AMP-dependent protein kinase, which boosts calcium influx through L-type channels and enhances calcium release from the sarcoplasmic reticulum. While essential for increasing myocardial contractility, excessive adrenergic activation can result in calcium overload. This overload leads to early and delayed afterdepolarisations, abnormal electrical impulses during or after repolarisation, which trigger arrhythmias and are linked to VES development [46,47]. 

Certain arrhythmic disorders, such as catecholaminergic polymorphic tachycardia and long QT syndrome (LQTS), are highly sensitive to abrupt adrenergic triggers, are responsible for the initiation of malignant ventricular arrhythmia or VES [48].

#### 3.1.6. Drugs as a Trigger

Medications can occasionally cause monomorphic VT via various mechanisms, including sodium channel activation or inhibition, calcium overload, β2-receptor stimulation, and coronary ischemia. Inhibition of sodium channels, especially by class IC antiarrhythmics like flecainide and propafenone, lowers conduction velocity and refractoriness, increasing re-entry and mortality risk in patients with fibrosis from past myocardial infarction or cardiomyopathy. Sodium channel activation delays repolarisation, enhancing ventricular automaticity. Calcium overload, seen with digoxin and methylxanthines toxicity, leads to afterdepolarisations and triggered activity. Other mechanisms include coronary steal caused by adenosine or dipyridamole, β2-stimulation (e.g., dobutamine, epinephrine), and drug-induced coronary vasospasm, myocarditis, or cardiomyopathy [2,49,50,51]. When drug-induced VT causes hemodynamic instability, immediate synchronised cardioversion is required. If the ventricular arrhythmia is stable, antiarrhythmics such as amiodarone and lidocaine can be administered. Both medications have proven effective for treating flecainide-induced VT. Procainamide may also be considered for patients with HFrEF who are not experiencing acute decompensation [2,49].

Over 1000 drugs can induce TdP by inhibiting the rapid delayed rectifier potassium current (IKr), causing prolonged action potential duration and increased susceptibility to early afterdepolarizations that can trigger TdP via phase 2 re-entry. Drugs like sotalol, dofetilide, ibutilidethioridazine, and erythromycin further extend ventricular action potential duration by enhancing late sodium current (INa-L), often via the phosphoinositide 3-kinase pathway. Variations in action potential duration across ventricular layers from differing ion current densities increase transmural dispersion of repolarisation, raising TdP risk. Unlike quinidine and sotalol, amiodarone does not amplify ventricular repolarisation dispersion, which may explain its lower TdP incidence [49,50,51,52]. To reduce the likelihood of TdP, clinical decision support tools have been developed. At the Mayo Clinic, a thorough computerised alert system for QT intervals is employed across the institution. This system assesses all electrocardiograms (ECGs) and alerts physicians when a patient’s corrected QT interval (QTc) is 500 milliseconds or longer [53]. The management of TdP involves defibrillating unstable or non-self-limiting arrhythmias. The causative medication must be discontinued without delay, and an infusion of 1–2 g of magnesium sulphate should commence, alongside corrections of potassium (K^+^) and calcium (Ca^2+^). In some instances, isoproterenol infusion or temporary pacing may be required. Mexiletine can be beneficial in managing TdP that is resistant to drug cessation and electrolyte replenishment [3,52,54].

### 3.2. The Substrate

The substrate refers to the fundamental structural and/or electrical irregularities in the heart, establishing a basis for the development of VES. The most common causes for an altered substrate are as follows:

∘ Fibrosis and scarring regions caused by previous myocardial infarction, chronic ischemia with consequent disruption of the normal electrical conduction pathways.
∘ Dilated or hypertrophic myocardium: structural changes associated with various cardiomyopathy types.
∘ Electrical remodelling: inherited or acquired changes in ion channel function, conduction velocity, or refractoriness [15].

#### 3.2.1. Altered Ventricular Substrate Associated with Heart Failure and Heart Failure Decompensation

The majority of patients with VES (77–94%) have an underlying structural heart disease, with advanced cardiomyopathy, either ischemic or non-ischemic, being the most prevalent condition [5,15]. Ischemic and non-ischemic cardiomyopathies represent critical substrates for the development of VES. In ischemic cardiomyopathy, the scarring and remodelling subsequent to myocardial infarction provide an arrhythmogenic substrate. Conversely, non-ischemic cardiomyopathies, including dilated or hypertrophic cardiomyopathy, contribute through heterogeneous fibrosis and abnormal electrical conduction. Both forms compromise the structural and electrical integrity of the myocardium, thereby facilitating the onset of ES and presenting unique challenges in clinical management. The most frequent non-ischemic cardiomyopathies at risk of VES are dilated cardiomyopathy, valvular heart disease, hypertrophic cardiomyopathy, and amyloidosis [55,56].

VES was associated in recent significant clinical trials with HF decompensation, and early mortality was almost primarily due to decompensated HF. Patients who died shortly after ES exhibited a rapid deterioration of systolic dysfunction and EF, which was the leading cause of mortality. The strongest predictors of mortality in these patients were an advanced degree of HF at the time of ES occurrence and any type of ICD, as therapy for a ventricular arrhythmic event, without difference between patients with devices implanted for primary or secondary prevention [57].

Cardiac resynchronisation therapy (CRT) responders have superior results compared to ICD therapy, resulting in a lower occurrence of ventricular electrical storms in patients with CRT, increasing the LVEF and consequently improving heart failure, positive impacts on the incidence of VES in both ischemic and non-ischemic cardiomyopathies. However, in some patients with CRT, left ventricular pacing was associated with the induction of VES and tended to occur early. A recent study indicates that CRT-induced arrhythmia rarely occurs more than three days after implantation [58]. In this case, immediate reprogramming or disabling of the CRT device is advised.

The most probable mechanisms of CRT-associated VES seem to be the following:

∘ Single-point biventricular pacing near a critical, slow-conduction left ventricular site, which can induce intramyocardial re-entry ventricular arrhythmias [58].
∘ The close proximity of the ventricular lead and the scarred myocardium can result in further scarring and local remodelling, which is potentially responsible for generating ventricular arrhythmia later during follow-up, after the device implantation [59].
∘ Increased transmural dispersion of ventricular repolarisation subsequent to the reversal of ventricular wall activation when pacing from the epicardial site in a structurally altered left ventricle, associated with QT interval prolongation and polymorphic ventricular tachycardia [60,61].
∘ Mechanical, caused by ventricular leads or triggered early after depolarisation-induced premature ventricular contractions [62].

##### Therapeutic Approach

The first step in the therapeutic approach for patients with structural heart disease is to control the acute cardiovascular or non-cardiovascular issue causing the electrical storm. Managing electrical storms in these patients starts with identifying and resolving the acute factors that lead to arrhythmic episodes. This critical initial step is essential for stabilising the patient and preventing the recurrence of ventricular arrhythmias. The most common triggers for ventricular electrical storms are myocardial ischemia and acute heart failure decompensation, which greatly disturb the fragile equilibrium of an affected heart’s electrical functions.

*Myocardial ischemia*, often due to coronary artery disease or acute coronary syndromes, creates an arrhythmogenic substrate by promoting ischemic injury, reducing myocardial perfusion, and increasing electrical heterogeneity. Revascularisation methods like percutaneous coronary intervention or coronary artery bypass grafting are crucial for restoring blood flow and reducing arrhythmogenic risks. Additionally, medical management with anti-ischemic drugs, antiplatelet agents, and anticoagulants helps prevent ischemia-induced arrhythmias.

*Acute heart failure decompensation*, frequently associated with fluid overload, increased myocardial stress, and neurohormonal activation, exacerbates ventricular dysfunction and electrical instability. Addressing this trigger involves optimising preload and afterload through diuretics, vasodilators, or inotropic agents, depending on the clinical scenario. Correcting hypoxia and electrolyte imbalances, such as hypokalemia or hypomagnesemia, is vital to stabilising myocardial electrophysiology. Non-pharmacological interventions, including mechanical circulatory support for patients with severe HF, can further aid in reversing decompensation and stabilising ventricular function.

Timely and tailored intervention for these acute triggers forms the cornerstone of ES management, reducing arrhythmia burden and improving overall cardiovascular outcomes [2,15,63,64,65].

*Electrolyte rebalance*. For hypokalemia, infusing potassium chloride at 20–40 mmol/L in 500 mL of saline is advisable, ensuring not exceeding 20 mmol/h. If higher rates are necessary, administering through a central venous catheter is recommended. The target plasma potassium level should be maintained within the 4.0–5.0 mmol/L range. Sometimes, refractory hypokalemia may be attributed to underlying hypomagnesemia, as magnesium deficiency can impair potassium reabsorption in the renal tubules and hinder potassium homeostasis. Correcting hypomagnesemia is often essential to effectively resolve persistent hypokalemia [66,67]. Two grams of magnesium sulphate intravenous bolus has been shown to reduce the incidence of TdP even with normal magnesium levels in patients with QT prolongation. The same treatment was ineffective in patients with polymorphic ventricular tachycardia and normal QT [68].

##### Sedative Therapy

First-line treatments for all patients include benzodiazepines like midazolam and short-acting opioid analgesics such as remifentanil. These medications help to minimise adrenergic overdrive and alleviate pain and discomfort during defibrillation while preventing negative inotropic effects (European Society of Cardiology Class I indication). Though propofol has shown effectiveness in treating refractory electrical storms by providing deep sedation and reducing arrhythmic triggers, it should be used with caution because of its considerable risk of negative inotropic effects. Despite these risks, propofol remains a valuable option in cases where benzodiazepines and opioids alone fail to achieve adequate suppression of arrhythmic episodes [3,63,69].

##### Antiarrhythmic Therapy

*Betablockers:* The suppression of adrenergic tone via betablockers still remains the first step of managing VES. While their beneficial therapeutic effects largely stem from their class effects, non-selective β1 and β2 blockers have more advantages in this context. In patients with chronic HF, ventricular remodelling leads to a downregulation of β1 receptors while β2 receptors are relatively preserved [70]. Furthermore, certain non-selective betablockers such as propranolol are lipophilic, allowing them to penetrate the central nervous system and block presynaptic adrenergic receptors. Propranolol (160 mg/d) has demonstrated efficacy in suppressing ventricular arrhythmias that are resistant to both metoprolol and amiodarone [71,72]. Both metoprolol and propranolol seem to have mild sodium channel blockade properties observed at exceptionally high doses [73]. Short-acting intravenous betablockers, such as esmolol, are beneficial in patients with hemodynamic compromise and heart failure with low ejection fraction [74]. The European Society of Cardiology (ESC) Class IIb recommendations advocate for autonomic modulation using intravenous beta-blockers, such as esmolol, in managing refractory electrical storm. The European Society of Cardiology suggests this approach as a late-stage intervention during resuscitation efforts to stabilise patients experiencing persistent arrhythmias [3]. The American College of Cardiology suggests using esmolol as an additional earlier therapeutic approach for VES, following metoprolol or propranolol [15]. Landiolol, another parenteral short-acting β1selective blocker, has been investigated as a potential treatment in VES. A clinical study by Miwa et al. showed that Landiolol given to patients in electrical storm refractory to defibrillation, epinephrine and amiodarone, successfully terminated ventricular arrhythmias in 80% of cases [75].

*Amiodarone:* Amiodarone is a class III antiarrhythmic and is the preferred treatment for VES linked to structural heart disease. It works by extending the ventricular refractory period and preventing re-entry when administered at loading doses of 800 to 1200 mg per day. Amiodarone may be continued up to a cumulative dose of 10–20 g, provided the patient remains free from ventricular arrhythmia for 48 h [2,3,15].

*Lidocaine*: Lidocaine, an antiarrhythmic drug classified as Class IB, is administered intravenously as a second-line treatment following amiodarone and is particularly effective in managing ischemic VT. It binds with greater affinity to inactivated Na+ channels that are found in partially depolarised myocytes and has the property to reduce electrical excitability and slow myocardial conduction. However, at elevated doses, it can lead to adverse effects on the central nervous system, including hallucinations, tremors, and seizures. To minimise the risk of these complications, it is advisable to monitor blood levels of lidocaine during treatment. The recommended doses for lidocaine are 1.0–1.5 mg/kg bolus, repeated 0.5–0.75 mg/kg every 5–10 min as needed up to 3 mg/kg, then 1–4 mg/min infusion [76,77].

*Procainamide:* Procainamide is usually regarded as a third-line antiarrhythmic agent for treating electrical storm. Its use is restricted because of toxicity concerns, the risk of accumulating its active metabolite, N-acetylprocainamide (NAPA), which can prolong the QT interval, especially in patients with kidney issues, and the lack of an oral option for long-term treatment. Procainamide p.o dose is 1000–4000 mg/d divided in multiple doses, while IV dose is 100–1000 mg load followed by 2–6 mg/min infusion [15,77].

*Mexiletine*, in doses of 450–900 mg/d po, can be used for the long-term management of recurrent ventricular arrhythmias in patients with HFrEF, in addition to amiodarone [67,78].

*Sotalol: This is administered in 160–320* mg in divided doses. In patients experiencing sustained VT, sotalol administered intravenously successfully terminated the arrhythmia within 15 min for 75% of patients [79]. However, Sotalol accounts for 17% of documented instances of polymorphic ventricular tachycardia [80]. Sotalol is not recommended for patients with renal impairment, reduced left ventricular ejection fraction, or advanced heart failure. In these cases, amiodarone and beta-blockers are generally preferred due to their safer profile and efficacy in managing arrhythmias [79,81].

Intravenous administration of amiodarone, lidocaine, procainamide, and sotalol presents various advantages and limitations for the acute termination of monomorphic ventricular tachycardia (VT) in patients with structural heart disease. Among these medications, procainamide exhibits the highest efficacy for terminating VT, followed by amiodarone and sotalol, both showing intermediate effectiveness, while lidocaine is the least effective. Procainamide’s notable efficacy has earned it a Class IIa recommendation for ending monomorphic VT, particularly in hemodynamically stable patients, whereas amiodarone and sotalol have Class IIb recommendations. However, procainamide is contraindicated in conditions like severe structural heart disease, decompensated HF, acute myocardial infarction, and advanced kidney disease, which are common in electrical storm patients. As a result, intravenous amiodarone is frequently preferred for those with VES associated with structural heart disease, particularly to support anti-tachycardia pacing or electrical cardioversion and to help prevent VT recurrence. Importantly, antiarrhythmic drugs can cause dose- and infusion rate-dependent hypotension due to mechanisms like α-1 blockade-mediated vasodilation and β-1 blockade-induced negative inotropy, with lidocaine showing the least risk of hypotension [2,3,15].

*Quinidine:* Current European and American guidelines recommend Quinidine as a class IIb indication in patients with recurrent polymorphic VT in patients unresponsive to other medications, along with autonomic modulation and mechanical circulatory support [3,15].

*Temporary pacing techniques:* Pacing may involve utilising a temporary transvenous pacing lead, a temporary external pacemaker linked to an active lead, or reprogramming an implanted device. Setting the pacing frequency at about 40% above the baseline rate and progressively increasing until the disappearance of PVCs and/or ventricular arrhythmia usually results in a positive response characterised by the absence of arrhythmia recurrence during continuous pacing [82]. Atrial or ventricular overdrive pacing can suppress abnormal ventricular automaticity by capturing the ectopic pacemaker, extinguishing its activity and inducing an exit block of the ectopic focus. In re-entry ventricular tachycardia, rapid pacing disrupts the re-entry circuit by modifying conduction rates, altering excitation pathways, shortening refractoriness, reducing dispersion of refractoriness, and promoting uniform tissue repolarisation. Decreasing the ventricular refractory period at higher pacing rates also lowers the risk of the R-on-T phenomenon [13].

*Autonomic tonus modulation*: The current possible interventions for autonomic tonus modulation are divided into three groups based on their specific primary target for modulation.

∘ The first category of interventions targets the intrinsic cardiac nervous system or myocytes, involving pharmacological inhibition with betablockers of the sympathetic nervous system, dissection, and glial modulation. Glial modulation has been researched in both the intrinsic cardiac nervous system and the stellate ganglia, linking the two [83].
∘ The second category (which includes cardiac sympathetic denervation, stellate ganglia block, thoracic epidural anaesthesia, and (auricular) vagal nerve stimulation) actively alters cardiac efferent pathways, reducing sympathetic outflow or enhancing parasympathetic tone. Thoracic epidural anaesthesia similarly impacts cardiac efferents and afferents, placing it at the intersection of the second and third categories [31].
∘ The third category (comprising spinal cord stimulation, carotid sinus stimulation, and renal denervation) primarily influences cardiac autonomic balance indirectly by modifying cardiac afferent activity, which in turn affects the efferent outflow mediated by integration centres along the cardiac neuraxis [84,85].

Thoracic epidural anaesthesia (TEA) has emerged as an effective, though temporary, intervention for managing VES. It involves the administration of a local anaesthetic—typically bupivacaine—into the thoracic epidural space through a catheter threaded beyond a Touhy needle. To ensure correct placement, clinicians verify the absence of blood or cerebrospinal fluid return. Treatment begins with a 1 mL bolus followed by a continuous infusion at 2 mL/h, with dosing adjusted based on arrhythmic suppression. TEA is frequently employed as a stabilising measure while more definitive therapies are planned and has demonstrated encouraging results in acute VES control [86,87].

Beyond TEA, autonomic modulation encompasses several techniques that target the sympathetic nervous system to dampen arrhythmogenic potential. Approaches such as percutaneous stellate ganglion block (SGB), thoracoscopic or open cardiac sympathetic denervation, and neuraxial modulation have gained traction, particularly in patients with structural heart disease. SGB has become a valuable bedside technique for the acute management of VES, a technique which is usually performed under ultrasound guidance using a 22-gauge, 2-inch spinal needle, advanced in-plane in a posterior-to-anterior direction to the anterior surface of the longus coli muscle, just behind the carotid artery. Care is taken to avoid vascular structures, and after confirming negative aspiration, 7 mL of bupivacaine is injected to achieve sympathetic blockade. SGB involves targeting the left or bilateral stellate ganglia with local anaesthetic, similar in principle to other autonomic modulation techniques. In the largest reported series of 30 patients undergoing urgent SGB for VES, 60% were free of VA within 24 h. Among those receiving frequent ICD therapies, SGB led to a dramatic 92% reduction in VA episodes, dropping from 26 ± 41 to 2 ± 4 over 72 h [64,88,89].

This procedure has demonstrated both safety and efficacy as a rapid intervention, particularly in the critical care setting, and continues to serve as a practical bridge while definitive therapies are considered.

##### Catheter Ablation

Catheter ablation was related to decreased recurrence of ventricular arrhythmia, fewer ICD discharges, decreased mortality and increased quality of life. Recent data on the impact of catheter ablation of VES derived from a multicentre study on 780 consecutive patients showed the superiority of catheter ablation over medical therapy alone, associated with a higher one-year and three-year survival, especially in patients over 70 years old and reduced LVEF (<35%) groups [90]. Data derived from a meta-analysis revealed that ablation therapy combined with ICD is more effective than standard ICD therapy alone in managing sustained VT caused by ischemic heart disease. The same research indicated better outcomes with ablation: both adjuvant and neoadjuvant ablation reduced VES, appropriate ICD interventions, heart failure-related hospital visits, and admissions related to cardiac issues [91]. A recent, large multicentre study using propensity score matching demonstrated that catheter ablation reduces VES recurrence and improves 1-year and 3-year survival rates compared to medical therapy alone. These benefits were consistent in older patients as well as in those with severely reduced LVEF [90].

While catheter ablation’s effectiveness in reducing the burden of arrhythmia is well established, long-term results following ablation of VT—especially in terms of mortality, recurrence and quality of life—were the focus of several pivotal studies like VANISH Trial and VANISH2 Extension. The original VANISH study showed that in patients with recurrent ischaemic cardiomyopathy and VT despite treatment with antiarrhythmic drugs, catheter ablation significantly reduced the composite endpoint of death, the VES and the appropriate ICD shocks compared to the increase in DAAs. In particular, patients who failed amiodarone treatment achieved the greatest benefit from ablation [92].

The latest VANISH2 study extended these findings by evaluating catheter ablation as first-line therapy. Over a median follow-up of 4.3 years, ablation reduced the risk of the composite primary endpoint by 25% compared to DAAs (50.7% versus 60.6%; HR 0.75, *p* = 0.03). Although all-cause mortality remained similar between groups, ablation was associated with less adequate ICD shocks, lower incidence of sustained VT below DCI detection thresholds, fewer non-fatal adverse events compared to DAAs. These results support the early use of ablation in selected patients, particularly those with ischemic substrates and high arrhythmic load [93].

The BERLIN VT study explored the timing of ablation by comparing preventive ablation at the time of ICD implantation versus deferred ablation after the third adequate ICD shock in patients with ischemic cardiomyopathy. Although the study was not designed to detect a mortality benefit, it was aimed to assess whether early intervention could reduce hospitalizations and arrhythmic events. The study highlighted several key aspects: preventive ablation was associated with a delay in the onset of VT-related events, patients who underwent early ablation experienced fewer symptomatic VT episodes, and the integration of telemonitoring and early rhythm control showed promise in improving patient outcomes. Even if definitive mortality data remain elusive in the BERLIN VT study, it showed the importance of timing in the VT ablation strategy, while supporting a more proactive approach among high-risk populations [94].

The use of mechanical circulatory support (MCS) during VT ablations has become increasingly recognised as a critical adjunct in the management of hemodynamically unstable patients. In people with advanced structural heart disease or severely reduced left ventricular function, induction and mapping of VT may precipitate deep hypotension or cardiogenic shock. Devices such as intra-aortic balloon pumps, Impella, and extracorporeal membrane oxygenation (ECMO) are commonly used to maintain perfusion and procedural stability of the final organs. The decision to initiate MCS is often guided by pre-procedural risk stratification, including factors such as ejection fraction, VT load, and prior hemodynamic tolerance. The incorporation of MCS not only facilitates more comprehensive mapping and ablation of substrates but can also improve procedural safety and long-term results in high-risk cohorts [95,96].

##### ICD Implantation

Implanting an ICD during a VES can lead to excessive ICD shocks, which is why it is usually saved for hospital discharge. After the remission of VES, the ICD implantation is recommended as a class I indication in patients with a life expectancy of over one year, with the mention of an increased risk of reoccurrence of the VES in the first two days after the implantation of the device [3,97]. The occurrence of the VES associated with the ICD implantation for primary prevention was reported to be 4 to 7% and 10 to 28% related to secondary prevention of sudden death. [98,99]. A proportion of 6.6% of patients implanted for both primary and secondary prevention of arrhythmic sudden death experience VES, and the main demographic and clinical associated characteristics were shown to be age over 65, a severely altered LVEF < 30%, the presence of chronic obstructive lung disease and also the absence of ACE inhibitor therapy [98]. ICD implantation is typically not advised for patients with persistent ventricular arrhythmias or advanced heart failure, unless they are candidates for a heart transplant or left ventricular assist device (LVAD) [15].

It is not yet clear if ventricular arrhythmia itself, repeated cardioversions, or both are responsible for increased mortality in patients with ICDs and VES. However, limited clinical and experimental research shows two directions potentially affecting the mortality rate in patients with ICD and heart failure- one is linked to intracellular calcium overload-induced decreased myocardial contractility during ventricular tachyarrhythmias and the other incriminates the implication of myocardial injury, inflammation and stunning, as well as the activation of the neurohormonal cascade in the deterioration of the myocardial contractility after ICD discharge [1,100].

##### ICD Reprogramming

Almost all centres proceed to deactivate the ICD direct current shock therapy in the case of VES. Some mechanistic studies assume that activation of adrenergic receptors and other direct consequences of ICD shocks promote arrhythmogenesis and decrease contractility [81]. OBSERVO-ICD study’s primary finding revealed that patients who developed VES following ICD implantation often had devices programmed with a more aggressive configuration, including lower VF detection thresholds, reduced detection times, and the exclusion of anti-tachycardia pacing during capacitor charging [16]. The primary goal of ICD reprogramming is to minimise ICD shocks by prioritising the termination of ventricular arrhythmias through ATP. Large-scale studies have demonstrated that extending detection duration (30–40 ventricular beats) and raising the heart rate detection threshold can effectively reduce ICD shocks without increasing the risk of mortality or syncope [74]. It is important to note that amiodarone may elevate the defibrillation threshold [101].

##### CRTp/CRTd Implantation

CRT is a class I guideline indication in patients with VES and heart failure NYHA II-IV, EF < 35%, QRS > 130 ms, or frequent ventricular pacing. Several mechanisms are implicated in reducing the ventricular arrhythmia burden in patients receiving CRT, and the first and seemingly the most important is reverse cardiac remodelling associated with increases in the LVEF. However, the failure rate of CRT responding is between 30% and 50%, mostly linked to pacing into a myocardial scar. The response rate was shown to be improved by multipoint left ventricular pacing and, more recently, by His bundle or left bundle branch (LBB) area pacing alone [102,103,104,105]. Multisite pacing significantly increases the rate of response to CRT, positively impacting the quality of life, the rate of morbidity and mortality and cardiac remodelling [106,107,108]. The multipoint left ventricular pacing has demonstrated superiority over single-site pacing in the case of CRT-associated ventricular arrhythmia and VES [102]. An experimental study in canine models showed that an increased number of left ventricular pacing sites was correlated to a significant reduction in the QT interval [38]. The recent I-CLASS study, which compared biventricular and LBB area pacing in terms of arrhythmic risk, showed a lower incidence of both ventricular and atrial arrhythmia for LBB pacing [109]. An exciting and relevant finding derived from the OBSERVO-ICD registry shows a 45% relative risk reduction for VES over a median time of 39 months in patients wearing a CRTd compared to ICD [22].

##### Stereotactic Radiation Therapy

Stereotactic body radiotherapy allows for the precise delivery of intense radiation to targeted tissue, minimising exposure to nearby structures. Recent research showed that stereotactic radiation therapy substantially decreased the VT burden during VES. Among patients without incessant VT, recurrences occurred in approximately half of the cases within the first six weeks. However, late single episodes of VT were linked to a prolonged cycle length [110].

##### ECMO and Ventricular Assist Devices

In patients suffering from refractory VES, extracorporeal membrane oxygenation (ECMO) has become an indispensable life-saving therapy when pharmacological and catheter-based interventions prove insufficient. By providing temporary circulatory and respiratory support, ECMO stabilises patients in hemodynamic crisis and creates a critical therapeutic window to track recovery, ablation or advanced mechanical circulatory support. Its indications include cardiogenic shock-supported VT/VF, recurrent post-cardiac arrhythmias, and cases where irreversible organ damage is imminent. Venoarterial ECMO is preferred in this context for its dual cardio-pulmonary support, usually deployed through femoral cannulation, with meticulous attention to anticoagulation and ventricular discharge. Early initiation—especially within 24 h—was associated with improved survival rates, particularly in post-myocardial infarction scenarios. As a bridge-to-decision tool, ECMO also allows for time for organ support and to evaluate neurological and transplant candidacy. Emerging data support its utility in reducing the arrhythmic load, facilitating catheter ablation in unstable patients, and improving long-term outcomes, even in higher-risk cohorts [111,112,113,114,115,116].

In conclusion, VES presents a critical challenge in cardiology, requiring a multifaceted therapeutic approach that integrates prompt stabilisation, advanced diagnostics and tailored management strategies. Initial care focuses on stabilising hemodynamics with antiarrhythmic drugs such as amiodarone, beta-blockers and sometimes lidocaine, while correcting underlying electrolyte imbalances. Sedation and mechanical support can be considered in serious cases, while, in parallel, evaluation of reversible causes—including ischaemia, electrolyte disturbances or drug toxicity—is essential. For recurrent episodes, catheter ablation targeting arrhythmogenic substrate has become a cornerstone of long-term management. Moreover, therapy with devices using ICDs plays a crucial role, supported by optimisation of programming to prevent shocks. Comprehensive care often involves collaboration between teams of electrophysiology, cardiology, and critical care to address the physical and emotional impact on patients (Table 3).

#### 3.2.2. Genetic Primary Arrhythmic Disorders

∘ Long QT syndrome

Long QT syndrome (LQTS) is the most common genetic electrical disorder with a dominant autosomal transmission and a reported prevalence of 1 in 2000, with a sudden death risk of 0.5% in asymptomatic patients and of around 5% in patients with syncope [117,118]. LQTS is characterised by a constant or intermittent prolongation of the QT interval on 12-lead ECG, with the specification that U waves should not be included in the measurement of the QT interval [119]. The actual accepted consensus for diagnosis uses two criteria: a corrected QT interval (QTc) equal to or over 480 ms or a risk score > 3 [3]. The risk score was first proposed by Schwartz et al. in 1993 [120]. It consists of major and minor criteria with corresponding numbers of points. The greater the sum of the points of the accomplished criteria, the greater the accuracy of diagnosis and the associated risk. The risk criteria used for establishing the diagnosis of LQTS consist of electrocardiographic findings, clinical history, and family history, which are summarised in Table 4 [120].

Individuals with LQTS require immediate, appropriate management and monitoring to prevent life-threatening arrhythmias. According to the European Society of Cardiology (ESC) guidelines for patients with ventricular arrhythmias, genetic testing and counselling are a class I recommendation for patients with clinically assessed LQTS [2]. There are three main subtypes depending on the specific mutation that account for over 90% of LQTS: LQTS1 (responsible gene KCNQ1), where ventricular arrhythmia is triggered by exercise; LQTS2 (KCNH2), where emotional stress triggers the condition; and LQTS3 (SCN5A), where sleep serves as the trigger. ECG findings can help distinguish between LQTS subtypes. They manifest as follows: LQTS1 is typically characterised by broad-based, peaked T waves contributing to QT prolongation; LQTS2 exhibits notched or bifid T waves; in LQTS3, the T wave appears late after a long ST segment, resulting in a prolonged QT interval [121].

In patients with LQTS1, exercise can trigger ventricular arrhythmias and VES because sustained β stimulation leads to the enhanced expression of the slow calcium current, Ca^2+^ L-type inward current. The slowly rectifying IKs potassium current does not counterbalance the calcium current because of a dysfunctional α-subunit of the potassium channel, critical for phase 3 of repolarisation. As a result, the action potential duration is prolonged. Sudden adrenergic surges were not found to induce this effect, likely because of the protective role of the intact fast-rectifying IKr current [13,122,123]. Previous research suggesting that exercise is the main trigger for sudden death events generated the concept that restraining any vigorous and sustained physical activity is potentially protective for LQTS-associated sudden death in both athletes and non-athletes. While beginning in 2015, the American Heart Association and American College of Cardiology relaxed the disqualification recommendations for competitive athletes with LQTS, the European Society of Cardiology recommendation remained restrictive due to a lack of prospective and comparative data [124]. In response to this dilemma, the very recent and the first prospective multinational LIVE-LQTS study showed a surprisingly low incidence, with no statistical difference in LQTS1-triggered ventricular arrhythmias linked to vigorous and non-vigorous exercise in appropriately treated patients. These findings can further guide discussions between patients and physicians and personalised decision-making regarding vigorous exercise, considering the overall expert assessment from a new perspective [125].

In LQTS2, arrhythmias were observed to be triggered by sudden arousal (such as loud noise or surprise associated with startle). The most popular explanation is that an abrupt adrenergic surge disrupts the balance between the APD-prolonging ICaL (Ca^2+^ L-type inward current) and dysfunctional IKr. Consequently, the APD is prolonged. Interestingly, more sustained adrenergic stimulation (such as during exercise) does not have the same effect due to the protective role of the intact countervailing IKs current [13,126].

In LQTS3, the bradycardia-triggered fatal ventricular arrhythmia is caused by the mutation of the SCN5A, which encodes the α-subunit of the sodium channel [127]. The mechanism of arrhythmia genesis at rest or during sleep at low heart rates is linked to enhanced sodium late current I_Na_, which fails to inactivate, prolonging the ADP, delaying the repolarisation and promoting the genesis of EADs [128,129].

The prolonged QT interval can lead to a specific type of ventricular tachycardia known as TdP, often initiated after an abrupt adrenergic stimulus. Clinically, TdP can cause symptoms such as syncope and can further degenerate into ventricular fibrillation and sudden cardiac death. A risk prediction model was developed by Mazzanti et al. and can currently be used as an online calculator (1-2-3-LQTS-Risk) to establish the 5-year risk of life-threatening arrhythmic events (sudden cardiac death, aborted cardiac death, hemodynamically unstable polymorphic ventricular tachycardia) based on specific parameters. The calculator uses the values of QT or QTc intervals measured in DII or V5 at a stable heart rate as close as possible to 60/min, RR interval and the associated genotype (LQTS1, LQTS2 or LQTS3) [130,131].

Non-selective beta-blockers (propranolol 80–320 mg/day, nadolol 40–120 mg/day) and mexiletine are recommended in LQTS patients to reduce arrhythmia risk [13]. Beta-blockers seem to be less effective in preventing ventricular arrhythmia in LQTS3 than in other LQTS [132].

Mexiletine is a sodium channel blocker with proven efficacy in patients with LQTS3 and also in patients with refractory ventricular arrhythmia. Mexiletine in a 3 *×* 400 mg/day loading dose followed by a 3 × 200 mg/day maintenance dose increases post-repolarisation refractoriness [133]. A recent study by Crotti et al. [134] evaluated mexiletine’s effect on human induced pluripotent stem cell-derived cardiomyocytes and showed a shortening of ADP of 113 ms and antiarrhythmic efficacy, being now considered a valid therapeutic option in patients with LQTS2. Ranolazine, an INaL current inhibitor, is recommended at doses between 750 and 2000 mg/day for arrhythmia prevention in LQTS3 but not in the other LQTS types [135].

Lidocaine, along with mexiletine, has a specific application in treating and preventing bradycardia-related polymorphic VT in the context of congenital or acquired long QT syndrome [76]. 

Temporary pacing at supranormal rates is the most widely used non-pharmacological strategy for controlling VES associated with LQTS [15]. Permanent pacing programmed to maintain a stable heart rate may be considered in patients with bradycardia-dependent ventricular arrhythmias [136].

Implantation of an ICD might be beneficial after the remission of VES [137]. In patients with ICD, deactivating shock, anti-tachycardia pacing (ATP), or both should be considered during VES [138].

The role of catheter ablation targeting triggers or substrate is limited in LQTS because of the need for significant clinical data.

A revolutionary LQTS3 therapy concept emerged from the recent experimental study data following the assessment of the effects of in vivo base editing of SCN5A in mice. In this study, researchers split the adenine base editor into two smaller parts and delivered them into the heart using adeno-associated virus serotype 9. The goal was to correct a pathogenic variant in the SCN5A gene. Effective amelioration of arrhythmia phenotypes in the mouse model with a QT correction rate exceeding 60% was obtained [129].

∘ *Catecholaminergic polymorphic ventricular tachycardia*

Catecholaminergic polymorphic ventricular tachycardia (CPVT) is a highly lethal form of inherited arrhythmogenic disorder characterised by life-threatening ventricular arrhythmias triggered by the physiological adrenergic state during stress or exercise, frequently diagnosed during exercise test. If left untreated, CPVT carries a significant risk, with mortality rates reaching as high as 31% by the age of 30 [139]. It is associated with mutations in the cardiac ryanodine receptor (RyR_2_), identified in 2001 by Priori et al. [140], responsible for the autosomal dominant form of the disease [141]. RyR_2_ is a Ca^2+^ channel that releases large quantities of calcium from the sarcoplasmic reticulum in response to the depolarisation of the cell membrane, initiating cardiac contraction [142]. The gain-of-function mutation induces a hyperreactivity of RyR2, which spontaneously releases Ca^2+^ from the sarcoplasmic reticulum through the receptors during adrenergic stimulation. As Ca^2+^ is released, a transient inward current occurs due to the sarcolemmal Na^+^-Ca^2+^ exchanger forcing out of the cell the released Ca^2+^. This newly produced electrogenic current depolarises the membrane during diastole, leading to DADs and then to ventricular arrhythmias [143,144]. Triggered activity initiating ventricular arrhythmia is frequently produced within the Purkinje cells.

Summarising, the interplay of mechanisms lying at the base of CPVT genesis includes the following [13,141]:

- Adrenergic stimulation;
- Calcium leak through the ryanodine receptor produces DAD;
- Triggered activity produced by DAD initiates ventricular arrhythmia.

Shortly after Priori et al. discovered RyR_2_, an autosomal recessive form of CPVT encoding the calsequestrin (CASQ2) gene was also identified. CASQ2 is a calcium-binding protein found in myocytes. Its primary role is to stabilise the free calcium concentration within the cell by acting as a calcium buffer, helping hold Ca^2+^ into the sarcoplasmic reticulum after muscle contraction. The protein plays a crucial role in excitation-contraction coupling and rhythm regulation [145].

A loss-of-function RyR_2_ mutation in relation to a recently described arrhythmic syndrome named RyR_2_ Ca^2+^ release deficiency syndrome (CRDS) was also associated with a high risk of sudden death. The syndrome is characterised by malignant ventricular arrhythmias occurring spontaneously and are not reproducible during stress test or other adrenergic stimuli [146]. The mechanism behind arrhythmogenesis in this syndrome is linked to RyR_2_ Ca^2+^ release deficiency, which is responsible for an increased store overload-induced Ca^2+^ release threshold. As a result, the abnormally large amounts of Ca^2+^ stored in the sarcoplasmic reticulum transients RyR_2,_ producing EADs and re-entry [147]. The use of the electrophysiological study for diagnostic purposes with a long burst-long pause-short coupling extra-stimulus protocol seems promising for inducing ventricular arrhythmia or for manifesting a specific broad and tall T wave and an abnormal QT interval prolongation on the first spontaneous beat after the cessation of pacing [148,149]. Data on the effectiveness of flecainide in controlling arrhythmias associated with CRDS is already emerging [150].

The electrocardiographic aspect of the ventricular arrhythmia, polymorphic and especially bidirectional ventricular tachycardia, may highly suggest CPVT. Catecholamines such as epinephrine and isoproterenol may increase the risk of an electrical storm by triggering a ventricular arrhythmia. They are sporadically used for diagnostic purposes when the exercise test fails to induce ventricular arrhythmias or is not possible (ESC Class IIa indication for diagnostic purposes) [3,151]. The diagnostic protocol recommends an initial infusion rate for epinephrine of 0.05 to 0.1 mcg/kg/min and afterwards, the progressive increase in the infusion rate by 0.05 mcg/kg/min up to a maximum allowable rate of 0.20 mcg/kg/min [152].

The management of CPVT-associated VES begins with electrical or pharmacological conversion or defibrillation, sedation, sympathetic blockade, and respiratory and circulat ory assistance if necessary [153]. The long-term management of clinically diagnosed CPVT typically involves the non-selective beta-blockers propranolol and nadolol titrated to the highest tolerated doses. Flecainide is also indicated in a conventional dose of 2–3 mg/kg/day or 200–400 mg/day in case of arrhythmia recurrence during beta-blocking treatment. However, its role in controlling VES is still unclear [154]. Verapamil can provide therapeutic benefits during VES in beta-blocker unresponsive cases [25]. In patients with CPVT, ICD shocks can be either ineffective or trigger VES. However, ICD implantation should be considered when arrhythmic events still occur while under treatment with the highest tolerated doses of beta-blocker and flecainide. Left cardiac sympathetic denervation is an effective therapeutic option in patients with CPVT associated with frequent ventricular arrhythmia in case of non-responsiveness to conventional therapy [3]. Isolated case reports and small studies showed the success of catheter ablation targeting trigger PVCs in controlling CPVT not controlled after beta-blocker treatment [155,156].

∘ *Brugada syndrome*

Brugada syndrome (BrS) is an inherited channelopathy expressed as the coexistence of typical ECG findings as more than 2 mm coved ST-segment elevation (a >2 mm J point elevation with a pseudo right bundle branch block pattern) followed by a negative T wave in at least one precordial derivation (from V1 to V3) for type 1 and a high risk of sudden death by ventricular malignant tachyarrhythmias [157]. Type 1 BrS is the only pattern considered critical for the diagnosis and can spontaneously manifest or be revealed by sodium channel blockade. The type 2 ECG pattern is also called saddle-back configuration. It is identified by ST-segment elevation in at least one V1 to V3 derivation, followed by a positive T wave in the same derivations. The type 3 BrS ECG pattern is characterised by a right derivation ST elevation of less than 1 mm, saddle-back or coved-shaped morphology [158]. BrS mutations are found in approximately 30% of cases, implying several gene mutations, the most prevalent of which is the mutation of SCN5A voltage-dependent sodium channels. It is interesting, however, that the majority of the patients do not manifest any mutation. Negative SCN5A results do not definitively exclude causal gene mutations. This is because, in general, investigations often do not explore the promoter region, cryptic splicing mutations, or the presence of gross rearrangements [159].

Moriata et al. showed that the right ventricular outflow tract is the main arrhythmogenic structure associated with BrS [56]. The presence of subepicardial cardiomyopathy with fibrosis patches of the extracellular matrix was also found in patients with BrS, suggesting an altered substrate for the associated VES on top of the inherited electric disease [160].

A recent study on the incidence and management of VES in patients with BrS revealed a summary of key findings:

- VES in BrS spontaneously resolves in one-third of cases;
- Recurrence of VES occurs in about 6.1% of the patients;
- Death related to VES is observed in 8.2% during follow-up [161].

An interesting study by Chakraborty et al. recently evaluated the dynamic ECG changes in response to the exercise test used for diagnostic and phenotyping purposes in patients with suspected BrS. Exercise-induced QRS duration prolongation is common in BrS, primarily due to delayed right ventricular activation. The study showed that the exercise-responsive electrocardiographic phenotype predicts a positive response to procainamide and may serve as a non-invasive screening tool to diagnose BrS before a drug challenge [162].

Regarding short-term management, Isoproterenol or Isoproterenol in combination with Quinidine is currently used to control acute-onset ventricular arrhythmia. Other drugs like Orciprenaline, Dysopiramide, Denopamide or Quinidine alone may be considered. The effectiveness of Isoproterenol, an arrhythmogenic medication in all other conditions, in interrupting VES was discussed in a 1999 work, which suggested the implication of an altered phase 2. Apparently, isoproterenol and isoprenaline decrease arrhythmogenesis by reducing the outward and increasing the inward calcium current, decreasing the duration of phase 2 of repolarisation. The indicated doses are Isoproterenol 1–2 μg intravenous bolus followed by 0.15–2.0 µg/min administered in continuous perfusion and Isoprenaline 1 to 2 µg bolus followed by 0.02–0.10 µg/kg/min and/or quinidine (300 to 1500 mg/day) administered orally or by gastric access [163]. Drugs such as mexiletine, lidocaine, magnesium sulphate and propofol were found to be ineffective in specific contexts [164]. Fever must be immediately treated by antipyretic medication; cooling can also be considered in cases of resistant fever. As for the long-term strategy of controlling VES, catheter ablation is an emerging alternative. Hyperpotasemia must be avoided in BrS because it can decrease sodium current and increase the risk of VES. The option of ICD implantation for primary prevention of sudden death is controversial in those patients. The electrophysiological study with programmed ventricular stimulation can be helpful in the risk stratification of BrS. Catheter ablation for controlling VES was first studied and introduced in the clinical practice by Haissaguerre et al., involving the ablation of local PVCs responsible for initiating ventricular arrhythmia.

Among patients with SCN5A mutations, there are some, often children, exhibiting a particular conducting Brugada phenotype, which manifests as the progressive broadening of the QRS complex at higher heart rates in a conduction-delay pattern. In these patients experiencing VES, Isoproterenol can be detrimental because further acceleration of the heart rate worsens intraventricular conduction delays, exacerbating electrical storms. Beta-blockers (Propranolol, Metoprolol) are indicated in these patients as they allow more time for proper conduction by lowering the heart rate on the one hand and reducing the sympathetic stimulation on the other [165].

A recently discovered inherited sudden cardiac death syndrome is linked to a risk haploid genotype that includes the dipeptidyl-peptidase 6 (DPP6) gene as a cause. This syndrome is not related to Brugada syndrome, still in the case of VES, it shares the same therapeutic management, including treating fever, intravenous administration of Isoproterenol, oral Quinidine and ICD implantation after the remission of VES [166].

A special attention should be paid to the acquired form of the ECG Brugada-like pattern induced most frequently by hyperkalemia because of its potential association with malignant arrhythmia and increased risk of all-cause mortality [167].

BrugadaDrugs.org is a non-profit project developed by the University of Amsterdam Academic Medical Center team and world experts in BrS. It aims to provide free, up-to-date information on safe drug use and can be accessed by BrS patients and medical caregivers worldwide [163].

∘ *Wolff–Parkinson–White syndrome*

Wolff–Parkinson–White (WPW) syndrome is a genetic, potentially arrhythmogenic condition with an incidence of 0.6–3:1000, characterised by the presence of one or more antegrade conducting accessory pathways (APs), also referred to as Kent bundles [168,169]. In addition, Kent bundles may also have retrograde conduction properties and actually, most of the manifest pre-excitation is associated with bidirectional conducting Kent bundles [170]. The APs are myocytes with altered electrical properties, serving as the structural foundation for pre-excitation by acting as an aberrant, additional bypass tract. The abnormal myocardial tissue caused by the failure to eliminate the remnants of atrioventricular connections during cardiogenesis spans the fibrous bridges between the atria and ventricles and has non-decremental conduction, unlike the atrioventricular node [171,172].

A ventricular electrical storm potentially linked to WPW syndrome can lead to sudden death, operating through an utterly distinct mechanism compared to those associated with all the other substrates. The concomitance of two factors, specific electrical properties of the Kent bundle and the presence of atrial fibrillation (AF) in WPW patients, is responsible for sudden death. The number of AF impulses conducted over the Kent bundle is directly related to its capacity to recover after the conduction of the previous impulse, namely the effective refractory period. Pre-excited AF conducted over an AP with an effective refractory period of less than 250 ms results in a very high ventricular rate and irregular, large QRS complex tachycardia with an R-R interval under 250 ms [173]. From pre-excited AF with very fast ventricular response to ventricular fibrillation, there is only one small step, as the hemodynamic collapse and consequent coronary arteries hypoperfusion will induce the vulnerability of the ventricular myocardium. This is the most common mechanism of sudden death in WPW syndrome.

Summarising, the properties of the AP/APs associated with the highest risk of sudden death are as follows:

∘ Effective refractory period of the AP < 250 ms
∘ Shortest pre-excited R-R interval of less than 250 milliseconds during AF
∘ Presence of multiple accessory pathways [174].

It is well known that patients with WPW and, even more likely, patients with multiple APs are predisposed to develop AF at a young age, not linked to acquired atrial changes like fibrosis or inflammation as encountered in the older aged population. Multiple explanations for the higher incidence of AF in patients with WPW have arisen over the years. Sharma et al. have shown that surgical methods and Haissaguerre et al. have demonstrated that catheter ablation both lead to a reduction in AF inducibility by eliminating the AP in patients with WPW syndrome [175,176,177]. Over time, it became increasingly evident that AP and its atrial insertions significantly contribute to initiating and maintaining AF through mechanisms specific to the WPW syndrome. The mechanisms implicated in AF genesis in this population are diverse. Sharma et al. reported a constant initiation of atrial tachycardia from the high right atrium regardless of the AP location, with consequent degeneration into AF, arriving mostly during atrial stimulation or reciprocating tachycardia. The electrophysiological properties associated with these findings were a longer PA interval, a shorter atrial effective refractory period and a shorter anterograde ERP of the AP [178]. Underlying electrophysiological features favouring AF were also described by Haissaguerre et al. in a study comparing the recurrence of AF after ablation within two distinct groups (patients with AF and patients with reciprocating tachycardia only). A shorter anterograde ERP of the AP increased atrial vulnerability and need for cardioversion were found in patients with induced AF as opposed to patients with reciprocating tachycardia only. One of the main findings was that successful catheter ablation of accessory pathways has been shown to prevent the recurrence of AF in 91% of patients [176].

Sudden cardiac death can, unfortunately, be the initial manifestation in patients with undiagnosed WPW syndrome. Notably, the risk of arrhythmic sudden death associated with WPW is greater during the first two decades of life [179]. A study by Di Mambro et al. found that young patients with ventricular pre-excitation have a similar risk of sudden death, regardless of whether they are symptomatic or asymptomatic [180]. Other studies also challenge the long-held belief, based on isolated research, that symptomatic patients are at greater risk, showing that half of the asymptomatic patients with persistent delta wave during the electrophysiological study manifested a high-risk profile of the AP [181]. Some interesting recent data show that the exercise test, classically used as a non-invasive tool for risk stratification in patients with ventricular pre-excitation, has a low negative predictive value and recommends electrophysiological study with isoproterenol infusion to accurately establish the AP-associated risk [181,182]. A multicenter study in children with WPW syndrome found similar results when evaluating the non-persistence of delta waves using ECG Holter monitoring and exercise test compared to EPS [183].

The emergency treatment of malignant arrhythmia associated with WPW consists of synchronised direct current electrical cardioversion in case of pre-excited AF with hemodynamic instability and prompt defibrillation in case of degeneration in ventricular fibrillation. In the acute setting, current guidelines recommend avoiding AV node-blocking agents (especially adenosine, non-dihydropyridine calcium blockers and digoxin) for the pharmacological treatment of pre-excited AF in stable patients, as they can induce a rapid ventricular response during AF [184,185]. Class IA (Ibutilide and Procainamide) and class IC antiarrhythmic drugs (Flecainide and Propafenone) are indicated in the acute treatment of pre-excited AF and AVRT. According to the ESC guidelines, the administration of Ibutilide and Procainamide has a Class IIa indication, level of evidence A, as long as Flecainide and Propafenone have a Class IIb indication, level of evidence B. One reason for the IIb indication of IC antiarrhythmic drugs in pre-excited AF is their impact on the atrioventricular node.

The long-term management relies on the catheter ablation of the AP, a low-risk, highly effective procedure with a success rate of more than 94%, while the recurrence rate after ablation ranges between 5 and 12% [186,187].

∘ *Early repolarisation syndrome*

The early repolarisation (ER) syndrome is a clinical diagnosis established in patients who have successfully been resuscitated from polymorphic ventricular tachycardia or ventricular fibrillation, without any underlying structural heart disease. The condition is indicated by the presence of an ER pattern defined as J point elevation of at least 1 mm in two or more contiguous leads of the 12-lead ECG, typically the infero-lateral leads [3]. There is a high risk of life-threatening ventricular arrhythmic events and sudden death associated with the ER pattern, but only when an arrhythmic event occurs in the context of this pattern, it is referred to as ER syndrome. The amplitude of the J-wave was found to be considerably higher in patients with VES compared to those without. Also, horizontal or descending ST segment and the inferolateral J-wave elevation are associated with increased arrhythmic risk [188,189]. ER pattern is a relatively common ECG finding present in approximately 5 to 10% of the population, with a higher prevalence among males, younger individuals and athletes. For now, it is typically considered benign in the vast majority of cases, although its link to cardiac arrhythmia still remains uncertain [190,191,192].

In a 2008 study, Haissaguerre et al. analysed 206 resuscitated patients with idiopathic ventricular fibrillation and found that ER was more prevalent in these patients (31%) compared to controls (5%). The patients with ER were more likely to be male and had a higher risk of recurrent ventricular fibrillation. Moreover, defibrillator monitoring confirmed an increased risk of arrhythmic events in patients with ER patterns [193].

ER syndrome shares significant clinical similarities with Brugada syndrome, including a higher prevalence in males, the typical age of onset for arrhythmic events, and the conditions under which episodes of ventricular arrhythmia or VES occur. These parallels suggest a common underlying pathophysiology, leading to their classification under J-wave syndromes. Genetic mutations affecting ion channel function lead to either an increase in outward K channels or a reduction in inward Na or Ca channels. The first identified mutation is in KCNJ8, resulting in a gain-of-function of IK−ATP. Other linked mutations include loss-of-function variants in CACNA1C, CACNB2, CACNA2D, SCN5A, and SCN10A, as well as gain-of-function mutations in ABCC9 and KCND3 [194].

In early repolarisation syndrome (ERS), as in Brugada syndrome, certain conditions, including bradycardia, beta-adrenergic blockade, vagal stimulation and fever, seem to promote arrhythmic disturbances, increasing susceptibility to cardiac events [13].

ICD implantation is recommended for patients with ER syndrome who have previously experienced a cardiac arrest, as class IB indication of the ESC Guidelines. Isoproterenol infusion has been shown to effectively suppress acute recurrent ICD discharges and electrical storm. Antiarrhythmic drugs that inhibit the transient outward potassium current can reduce the risk of VF, with quinidine demonstrating a significant decrease in VF recurrence in a multicenter study. Additionally, phosphodiesterase-3 inhibitors, including cilostazol and milrinone, have been associated with lower VF recurrence rates. Catheter ablation, particularly targeting PVCs triggers from the Purkinje system, has shown high success rates (87–100%), making it an effective intervention for patients with drug-resistant VF [3,195,196].

∘ *Short QT syndrome*

Short QT syndrome (SQTS) is a rare primary electrical disease introduced as an individual entity in 2000 by Gussak et al. [197]. SQTS is diagnosed when QTc < 320 ms with or without arrhythmia or when QTc is between 320 ms and 360 ms in the presence of arrhythmic syncope. Depending on the genetic basis, currently, there are several known forms of SQTS, all with autosomal dominant transmission:

▪ SQTS1 is associated with the gain-of-function mutation KCNH2 of the delayed rectifying potassium current rapid component (IKr). The result is that the inactivation of IKr occurs at a much more positive voltage than the normal, typical voltage for the cardiac action potential, between −90 and +30 mV. When the inactivation voltage is shifted by +90 mV, the IKr is not inactivated during normal action potential, interfering with the repolarisation phase and leading to ventricular arrhythmia [198].
▪ SQTS2 is linked to the gain-of-function mutation KCNQ1 of the delayed rectifying potassium current (IKs), which induces an accelerated activation resulting in the opening of the IKs channels at a −20 mV more negative voltage than normal. The earlier activation of the current caused by the opening of the channels at a more negative current induces an earlier repolarisation phase implicated in ventricular arrhythmia generation [199].
▪ SQTS3 is linked to another gain-of-function mutation of another gene, KCNJ2, encoding the Kir2.1 protein, a component of the inward rectifying potassium current channel (IK1). This mutation is associated with extremely short QT intervals of 315–320 ms. IK1 is a key current in maintaining a normal resting membrane potential, and also plays a role in the final phase of repolarisation in the cardiac cells. Typically, at potentials between −75 and −45 mV, an increase in the outward IK1 results in potassium ions leaving the cell, stabilising the membrane’s resting potential and making it more likely to be spontaneously depolarised. In patients with KCNJ2 mutation, the IK1 allows the flow out of a larger number of K ions than normal, accelerating the repolarisation phase of the cardiac action potential, destabilising the membrane’s resting potential and predisposing to ventricular fibrillation [200,201,202].
▪ SQTS4 is linked to the mutation of the CACNA1C gene, which encodes the alpha 1 subunit of IK1, resulting in the gain of function, increasing the outward IK1 and enhancing the repolarisation phase by shortening the action potential duration [203].
▪ SQTS5 is caused by a CACNB2 gene mutation encoding the beta-2b subunit of IK1, shortening the action potential duration and decreasing refractoriness.

The risk of sudden death by malignant ventricular arrhythmias is high and can be the first manifestation of SQTS. The association with atrial fibrillation (AF) is up to 70%, and in half of the patients, the initial manifestation was AF. This might be a sufficient reason to screen for SQTS in young patients with AF. The invasive electrophysiological study seems to be a trusted screening tool, as in 90% of the patients, ventricular fibrillation was induced at programmed ventricular stimulation. Another important finding during the electrophysiological study was unusually short effective atrial and ventricular effective refractory periods [204]. Although quinidine is not included in the European Society of Cardiology guidelines for treating atrial fibrillation, it is referenced nearly 40 times in the 2022 European guidelines for ventricular arrhythmias and 20 times in the 2017 Heart Rhythm Society guidelines, including for treating SQTS [3,205,206]. A non-randomised study by Gaita et al. compared four antiarrhythmic drugs from different classes: hydroxyquinidine (IA), flecainide (IC), sotalol (III) and ibutilide (III). The only drug that showed a positive effect at a dose of 250 mg three times daily was hydroxyquinidine, associated with significant QT prolongation, increasing the ventricular effective refractory period and decreasing the inducibility of ventricular fibrillation [207].

Isoproterenol may be used in SQTS-associated VES. However, the evidence is limited [208].

According to the ESC Guidelines, ICD implantation in SQTS is a class IC indication for patients with documented VT or among arrhythmic sudden death survivors and an IIa indication (should be considered) for patients with arrhythmic syncope and a class IIb indication (may be considered) for patients with a QT interval between 320 and 360 ms who have a family history of sudden death at ages under 40 years [3].

∘ *Arrhythmogenic right ventricular cardiomyopathy*

Arrhythmogenic right ventricular cardiomyopathy (ARVC) is a genetic myocardial condition impacting the right ventricle, left ventricle, or both. The 2019 Heart Rhythm Society guidelines identify 12 ARVC-associated genes. While most cases involve mutations in desmosomal genes, non-desmosomal variants also occur. However, the strongest scientific evidence supports the role of desmosomal gene mutations, whereas connections with non-desmosomal proteins require further investigation [209]. The disease is characterised by fibrofatty replacement of the myocardium, leading to systolic ventricular dysfunction and potentially life-threatening ventricular arrhythmias, which can result in SCD, particularly in young individuals and athletes [210]. Postmortem analysis of young athletes who experienced SCD have identified ARVC in 10–15% of cases [211]. The progression of ARVC is primarily characterised by ventricular electrical instability, which can result in arrhythmic sudden cardiac death, particularly in young individuals and athletes. In later stages of the disease, the deterioration of right ventricular muscle and involvement of the left ventricle can lead to right-sided or biventricular heart failure [212]. Ventricular fibrillation tends to occur more often in younger, physically active individuals compared to older patients. Additionally, research has shown a higher VF prevalence in those carrying multiple genetic mutations, suggesting a possible link between genetic complexity and arrhythmia risk [213].

Pharmacological treatment for ARVC focuses on managing heart failure, preventing thromboembolic events and controlling arrhythmias. The antiarrhythmic medications include betablockers, which are often used prophylactically for ARVC patients with ventricular arrhythmias, while flecainide can serve as an additional therapy for recurrent cases. Current research on antiarrhythmic drugs in ARVC has yielded mixed results. One study found sotalol effective for VT, while amiodarone was not shown to be superior. Another study reported that betablockers, including sotalol, provided limited protection against VT, whereas amiodarone significantly reduced its occurrence [214,215,216].

Catheter ablation has been proven effective in reducing the frequency of VT episodes and preventing recurrent electrical storms in patients with ARVC. Current guidelines and expert consensus endorse its use as a valuable treatment option [217,218]. Recent research shows that biventricular involvement is linked to faster VT, higher inducibility and more widespread arrhythmogenic substrates. The left ARVC appears to be associated with left-sided VTs, which tend to be more severe than right-sided VTs and are linked to a significant decline in left ventricular systolic function. Catheter ablation provides comparable short and long-term VT control in patients with and without biventricular involvement. Predictors of VT recurrence after ablation include younger age, reduced right ventricular ejection fraction and partial or unsuccessful procedures [219].

Regarding ICD implantation, although randomised studies on secondary prevention are lacking, the high occurrence of appropriate ICD interventions in patients with fast VT, VES and VF episodes following catheter ablation or poorly tolerated VT strongly suggests a survival benefit from ICD implantation [3,220].

∘ *Idiopathic ventricular fibrillation*

Idiopathic ventricular fibrillation (IVF) is an uncommon cause of sudden cardiac arrest with no identifiable substrate after extensive diagnostic testing (toxicologic, channelopathies, metabolic, respiratory), especially in the young [221]^.^

Recent research has identified a high occurrence of microstructural abnormalities, which seem to be the foundation for ventricular fibrillation re-entries. These subtle changes, often found in small, isolated regions of slow conduction in both myocardium and epicardium, can only be detected through detailed high-density endo and epicardial mapping. The nature of changes points towards various genetic and acquired conditions such as cell connectivity or tissue structure, cardiomyopathies, fatty infiltration and myocarditis. Associated Purkinje system abnormalities associated with these changes play a significant role in the occurrence of IVF [222]. Various arrhythmic patterns linked to Purkinje ectopy, such as short coupling and multifocal PVCs have been described in association with IVF [223].

After ICD implantation, pharmacotherapy is used to reduce ICD interventions or to antagonise the arrhythmic vulnerability in patients with frequent PVCs, for example, in patients who either lack a suitable substrate for catheter ablation or prefer to avoid invasive procedures. Beta-blockers are recommended for patients experiencing arrhythmias during exertion or stress. Verapamil is highly effective for managing frequent Purkinje-related triggers and polymorphic VT and is more reliable as an immediate short-acting intravenous treatment rather than for long-term oral use. Quinidine has also demonstrated benefits in IVF, significantly decreasing ICD shocks, while interruptions in treatment are associated with recurring arrhythmic events [3,224,225].

## 4. Conclusions

Ventricular electrical storms are a life-threatening cardiac emergency characterised by recurrent episodes of ventricular arrhythmias, presenting significant challenges for both patients and physicians. Effective management requires a mechanism-based approach that integrates knowledge of electrophysiological disturbances, autonomic influences, and underlying structural or genetic factors contributing to electrical instability. Over the years, advancements in treatment strategies have provided a multi-faceted framework for stabilising and preventing these arrhythmic events. Pharmacologic therapies targeting ion-channel dysfunction, such as beta-blockers, antiarrhythmic agents, and neuromodulatory drugs, form the cornerstone of acute and long-term management. In patients refractory to medical therapy, catheter ablation has emerged as a powerful tool in efforts to eliminate arrhythmogenic substrates and reduce arrhythmic burden. Beyond traditional interventions, neuromodulation strategies, including stellate ganglion blockade and sympathetic denervation, offer promising alternatives, especially for individuals with recurrent and intractable arrhythmias. Moreover, ICDs remain essential for patients at high risk of sudden cardiac death, providing life-saving interventions during ventricular electrical storms.

The evolution of genetic and molecular research continues to refine our understanding of ventricular electrical storm pathophysiology, enabling the use of precision medicine approaches tailored to individual patient profiles. Emerging technologies, including artificial intelligence-driven predictive models and wearable cardiac monitoring devices, further contribute to early detection and prevention.

Despite significant progress in the acute and long-term management of VES, high morbidity and mortality indicate the need for further innovation. Emerging strategies cover pharmacological, interventional, and computational areas, providing promising future solutions for risk prediction and therapeutic refinement.

Ongoing Clinical Studies and Investigational Therapies: Several multicentre studies are ongoing to evaluate catheter ablation, autonomous modulation, and the timing of mechanical circulatory support in VES. These studies aim to clarify optimal sequencing and identify subgroups of patients most likely to benefit from aggressive early intervention. In addition, studies exploring non-selective blockers, stellate ganglion blockers, and ECMO-assisted ablation expand the evidence base for rescue therapies.

Novel Pharmacological Agents: Selective ion channel modulators gain traction as targeted antiarrhythmic agents, including late sodium current inhibitors and IKr stabilisers, which can reduce repolarization dispersion and suppress triggered activity, connexin-43 modulators that increase junction integrity and reduce heterogeneity of the arrhythmic substrate, and TRPV1 antagonists, which target related reflex pathways involved in sympathetic overload and arrhythmogenesis.

Artificial Intelligence and Predictive Modelling: AI is poised to revolutionise risk stratification and the early detection of VES by machine learning algorithms trained on electro-anatomical mapping. ICD telemetry and clinical variables demonstrated superior predictive accuracy compared to conventional scoring systems, multimodal deep learning models, which integrate imaging, ECG, and electronic health records, aiming to forecast arrhythmic disorders.

Ultimately, a multidisciplinary approach, combining expertise from cardiology, electrophysiology, neurology, and emerging technologies, is essential in improving long-term outcomes. By bridging fundamental science with clinical applications, ventricular electrical storm management continues to advance toward personalised, mechanism-based treatments, ensuring more effective and sustainable results for affected patients.

## Figures and Tables

**Table 1:** Cardiac and extracardiac risk factors for ventricular electrical storm, incidence and mortality associated with ventricular storm. VES—ventricular electrical storm, LVEF—left ventricular ejection fraction, VA—ventricular arrhythmia, HF—heart failure, BMI—body mass index, ACM—arrhythmogenic cardiomyopathy, ICDs—implantable cardioverter defibrillators, CRT-D—Cardiac resynchronization therapy with a defibrillator, RV—right ventricle.

Authors	Risk Factors	Study	VES Incidence	Mortality
Guerra et al. [22]	Lower LVEF Prolonged QRS duration Previous VA episodes The use of antiarrhythmic class I	Multicenter registry (5 Italian arrhythmia centres), 2010–2012, >1000 patients ≥18 years with ICD/CRT-D implantation; propensity score matching for ICD vs. CRT-D groups (Guerra et al.).	10–28% in patients with secondary prevention ICDs	Increased risk of death and combined risk of death, heart transplantation and hospitalisation for HF
Ninni et al. [20]	Age Lower LVEF RV dysfunction Haemoglobin level Use of catecholamines at admission	Single-centre retrospective study, 2015–2020, 253 patients hospitalised for electrical storm in the ICU; median age 66 years, 64% with ischemic cardiomyopathy, 37% required catecholamines at admission.	10–58% for secondary prevention, 4–7% for primary prevention	One-year mortality of 34%, mostly driven by HF
Zhai et al. [23]	High BMI Extensive T-wave inversion	Single-centre study, Fuwai Hospital, Beijing, 2005–2020, 88 ACM patients with ICDs; median follow-up 4.0 years	21.6% in ACM patients with ICDs	No independent increase in cardiac mortality

**Table 2:** The phases of the standard cardiac action potential with the respective ionic movement.

Phase 0 (depolarisation)	Rapid influx of Na^+^ ions over the voltage-activated channels
Phase 1 (initial repolarisation)	Short repolarisation caused by transient K^+^ efflux
Phase 2 (plateau)	Continuous depolarisation due to a balanced K^+^ efflux and Ca influx
Phase 3 (repolarisation)	Closure of Ca^2+^ channels and persistence of K^+^ efflux leads to repolarisation
Phase 4 (resting potential)	The influx of K^+^ mediated by the inward rectifier K^+^ channel results in a stable resting potential of the membrane

**Table 3:** Summary of therapeutic strategies in VES management.

Strategy	Mechanism of Action	Evidence Level/Guideline Class	Clinical Considerations
**Acute trigger control**	Resolves underlying ischemia, HF decompensation	ESC: Class I	First step in all VES cases: urgent revascularization and volume optimisation as needed
**Electrolyte correction**	Stabilises cardiac membrane potentials	ESC: Class I	Maintain K^+^ at 4.0–5.0 mmol/L; MgSO_4_ bolus for QT prolongation; avoid rapid infusion
**Sedation (e.g., midazolam, remifentanil)**	Reduces sympathetic drive and arrhythmogenic triggers	ESC: Class I	Effective for autonomic suppression; propofol use cautioned due to negative inotropic effects
**Beta-blockers**	Adrenergic suppression via β1/β2 blockade	ESC: Class I–IIb (esmolol, landiolol)	Propranolol preferred in CNS-penetrating cases; esmolol for low EF and shock; consider lipophilicity
**Amiodarone**	Blocks Na^+^, K^+^, Ca^2+^ channels; prolongs APD	ESC: Class I	Preferred in structural heart disease; monitor cumulative dose and defibrillation threshold
**Lidocaine**	Inhibits inactivated Na^+^ channels in ischemic myocardium	ESC: Class IIb	Second-line in ischemic VT; short half-life; dose titration essential
**Procainamide**	Na^+^ channel blocker; NAPA prolongs QT	ESC: Class IIa (VT termination)	Avoid in renal dysfunction; highest VT termination rate; toxicity limits long-term use
**Mexiletine**	Oral Na^+^ channel blocker; suppresses ectopy	ESC: Class IIb	Used adjunctively with amiodarone in HFrEF; long-term suppression of arrhythmias
**Sotalol**	Beta-blocking + APD prolongation	ESC: Class IIb	Intermediate efficacy; watch for QT prolongation
**Quinidine**	Class Ia antiarrhythmic; prolongs QT	ESC and ACC: Class IIb	For polymorphic VT unresponsive to other agents; limited use due to side effect profile
**Temporary pacing**	Overdrive suppression of ventricular ectopy	ESC: Class IIb	Useful for arrhythmias triggered by bradycardia or PVCs
**Thoracic Epidural Anaesthesia**	Sympathetic blockade via neuraxial anaesthesia	ESC: Class IIb	Short-term control; requires skilled placement and monitoring; bridge to ablation
**Stellate Ganglion Block (SGB)**	Suppresses sympathetic cardiac input	ESC: Class IIb	Bedside-accessible; ultrasound guided; 60–92% success in VT suppression
**Cardiac Sympathetic Denervation**	Reduces afferent sympathetic tone	ESC: Class IIa–IIb	Surgical or thoracoscopic; effective in inherited arrhythmia syndromes and structural disease
**Catheter ablation**	Substrate modification and re-entry circuit elimination	ESC: Class I (structural VT), VANISH trial	Definitive for recurrent VT; improves survival and quality of life; guided by electroanatomic mapping
**ICD reprogramming**	Minimises shock burden; favours ATP	ESC: Expert consensus	Lengthen detection time; raise VF thresholds; deactivate shock delivery during VES episode
**ICD implantation**	Detects and terminates malignant arrhythmias	ESC: Class I (post-VES recovery)	Avoid implantation during VES; reassess after stabilisation
**CRT implantation**	Cardiac resynchronization; reverses remodelling	ESC: Class I	Recommended in VES with HF (EF < 35%, QRS > 130 ms); response improved by LBB/HB pacing
**Stereotactic radiotherapy**	Targeted ablation of arrhythmic focus	Investigational/Emerging	Non-invasive option under evaluation; precise, tissue-sparing technique
**ECMO (VA-ECMO)**	Mechanical support for cardiac and pulmonary function	ESC: Expert Consensus/Emerging	Life-saving bridge in cardiogenic shock; facilitates ablation or transplant evaluation; survival benefit if early

**Table 4:** Risk assessment criteria for the diagnosis of LQTS.

Schwartz Diagnostic Score for Long QT Syndrome (LQTS)		
Category	Criteria	Points
ECG Findings	QTc ≥ 480 ms	3
	QTc 460–479 ms	2
	QTc 450–459 ms	1
	QTc ≥ 480 ms (4th min of recovery post-exercise)	1
	Torsade de pointes	2
	T-wave alternans	1
	Notched T-waves in ≥3 leads	1
	Low heart rate for age	0.5
Clinical History	Syncope (stress-induced)	2
	Syncope (non-stress-induced)	1
	Congenital deafness	0.5
Family History	Definitive LQTS in the family	1
	Sudden death (age < 30 years) in the family	0.5
Genetic Findings	Pathogenic mutation	3.5

LQTS—long QT syndrome; ECG—electrocardiographic; QTc—corrected QT interval; HR—heart rate (After Schwartz et al. [120]).

## References

[1] Sagone A. (2015). Electrical Storm: Incidence, Prognosis and Therapy. J. Atr. Fibrillation.

[2] Al-Khatib S.M., Stevenson W.G., Ackerman M.J., Bryant W.J., Callans D.J., Curtis A.B., Deal B.J., Dickfeld T., Field M.E., Fonarow G.C. (2018). 2017 AHA/ACC/HRS Guideline for Management of Patients with Ventricular Arrhythmias and the Prevention of Sudden Cardiac Death: Executive Summary: A Report of the American College of Cardiology/American Heart Association Task Force on Clinical Practice Guidelines and the Heart Rhythm Society. Circulation.

[3] Zeppenfeld K., Tfelt-Hansen J., De Riva M., Winkel B.G., Behr E.R., Blom N.A., Charron P., Corrado D., Dagres N., De Chillou C. (2022). 2022 ESC Guidelines for the Management of Patients with Ventricular Arrhythmias and the Prevention of Sudden Cardiac Death. Eur. Heart J..

[4] Elsokkari I., Parkash R., Tang A., Wells G., Doucette S., Yetisir E., Gardner M., Healey J.S., Thibault B., Sterns L. (2020). Mortality Risk Increases with Clustered Ventricular Arrhythmias in Patients with Implantable Cardioverter-Defibrillators. JACC Clin. Electrophysiol..

[5] Guerra F., Shkoza M., Scappini L., Flori M., Capucci A. (2014). Role of Electrical Storm as a Mortality and Morbidity Risk Factor and Its Clinical Predictors: A Meta-Analysis. Europace.

[6] Geraghty L., Santangeli P., Tedrow U.B., Shivkumar K., Kumar S. (2019). Contemporary Management of Electrical Storm. Heart Lung Circ..

[7] Gama F., Ferreira J., Carmo J., Costa F.M., Carvalho S., Carmo P., Cavaco D., Morgado F.B., Adragão P., Mendes M. (2020). Implantable Cardioverter–Defibrillators in Trials of Drug Therapy for Heart Failure: A Systematic Review and Meta-Analysis. J. Am. Heart Assoc..

[8] Eifling M., Razavi M., Massumi A. (2011). The Evaluation and Management of Electrical Storm. Tex. Heart Inst. J..

[9] Tsuji Y., Heijman J., Nattel S., Dobrev D. (2013). Electrical Storm: Recent Pathophysiological Insights and Therapeutic Consequences. Basic Res. Cardiol..

[10] O’Shea C., Winter J., Kabir S.N., O’Reilly M., Wells S.P., Baines O., Sommerfeld L.C., Correia J., Lei M., Kirchhof P. (2022). High Resolution Optical Mapping of Cardiac Electrophysiology in Pre-Clinical Models. Sci. Data.

[11] Sutanto H., Heijman J. (2022). Integrative Computational Modeling of Cardiomyocyte Calcium Handling and Cardiac Arrhythmias: Current Status and Future Challenges. Cells.

[12] Colman M.A., Alvarez-Lacalle E., Echebarria B., Sato D., Sutanto H., Heijman J. (2022). Multi-Scale Computational Modeling of Spatial Calcium Handling From Nanodomain to Whole-Heart: Overview and Perspectives. Front. Physiol..

[13] Lenarczyk R., Zeppenfeld K., Tfelt-Hansen J., Heinzel F.R., Deneke T., Ene E., Meyer C., Wilde A., Arbelo E., Jędrzejczyk-Patej E. (2024). Management of Patients with an Electrical Storm or Clustered Ventricular Arrhythmias: A Clinical Consensus Statement of the European Heart Rhythm Association of the ESC—Endorsed by the Asia-Pacific Heart Rhythm Society, Heart Rhythm Society, and Latin-American Heart Rhythm Society. EP Eur..

[14] Exner D.V., Pinski S.L., Wyse D.G., Renfroe E.G., Follmann D., Gold M., Beckman K.J., Coromilas J., Lancaster S., Hallstrom A.P. (2001). Electrical Storm Presages Nonsudden Death: The Antiarrhythmics versus Implantable Defibrillators (AVID) Trial. Circulation.

[15] Jentzer J.C., Noseworthy P.A., Kashou A.H., May A.M., Chrispin J., Kabra R., Arps K., Blumer V., Tisdale J.E., Solomon M.A. (2023). Multidisciplinary Critical Care Management of Electrical Storm: JACC State-of-the-Art Review. J. Am. Coll. Cardiol..

[16] Guerra F., Palmisano P., Dell’Era G., Ziacchi M., Ammendola E., Bonelli P., Patani F., Cupido C., Devecchi C., Accogli M. (2016). Implantable Cardioverter-Defibrillator Programming and Electrical Storm: Results of the OBSERVational Registry On Long-Term Outcome of ICD Patients (OBSERVO-ICD). Heart Rhythm..

[17] Pedersen C.T., Kay G.N., Kalman J., Borggrefe M., Della-Bella P., Dickfeld T., Dorian P., Huikuri H., Kim Y.-H., Knight B. (2014). EHRA/HRS/APHRS Expert Consensus on Ventricular Arrhythmias. Europace.

[18] Elsokkari I., Sapp J.L. (2021). Electrical Storm: Prognosis and Management. Prog. Cardiovasc. Dis..

[19] Prystowsky E.N., Padanilam B.J., Joshi S., Fogel R.I. (2012). Ventricular Arrhythmias in the Absence of Structural Heart Disease. J. Am. Coll. Cardiol..

[20] Ninni S., Layec J., Brigadeau F., Behal H., Labreuche J., Klein C., Schurtz G., Potelle C., Coisne A., Lemesle G. (2022). Incidence and Predictors of Mortality after an Electrical Storm in the ICU. Eur. Heart J. Acute Cardiovasc. Care.

[21] Wen Z., Tang W., Wu Z., Shui X., Zheng Z. (2024). Renal Outcomes and Associated Contributing Factors in Patients with Acute Myocardial Infarction—A Retrospective Cohort Study. J. Biol. Regul. Homeost. AGENTS.

[22] Guerra F., Palmisano P., Dell’Era G., Ziacchi M., Ammendola E., Pongetti G., Bonelli P., Patani F., Devecchi C., Accogli M. (2018). Cardiac Resynchronization Therapy and Electrical Storm: Results of the OBSERVational Registry on Long-Term Outcome of ICD Patients (OBSERVO-ICD). Europace.

[23] Zhai L., Hu Y., Li X., Zhang X., Gu Z., Zhao Z., Yang X. (2021). Incidence, Predictors and Clinical Impact of Ventricular Electrical Storm in Arrhythmogenic Cardiomyopathy Patients with an Implantable Cardioverter–Defibrillator: A Single-Center Report with Medium-Term Follow-Up. Int. J. Gen. Med..

[24] Farré J., Wellens H.J. (2004). Philippe Coumel: A Founding Father of Modern Arrhythmology. Europace.

[25] Maruyama M. (2014). Management of Electrical Storm: The Mechanism Matters. J. Arrhythmia.

[26] Trohman R.G. (2024). Etiologies, Mechanisms, Management, and Outcomes of Electrical Storm. J. Intensive Care Med..

[27] Gaztañaga L., Marchlinski F.E., Betensky B.P. (2012). Mechanisms of Cardiac Arrhythmias. Rev. Esp. Cardiol..

[28] Tomaselli G., Roden D.M. (2005). Chapter 1—Molecular and Cellular Basis of Cardiac Electrophysiology. Electrophysiol. Disord. Heart.

[29] Hanna P., Ardell J.L., ShivkumarKalyanam K. (2020). Cardiac Neuroanatomy for the Cardiac Electrophysiologist. J. Atr. Fibrillation.

[30] Ardell J.L., Armour J.A. (2016). Neurocardiology: Structure-Based Function. Comprehensive Physiology.

[31] van Weperen V.Y.H., Vos M.A., Ajijola O.A. (2021). Autonomic Modulation of Ventricular Electrical Activity: Recent Developments and Clinical Implications. Clin. Auton. Res..

[32] Herren A.W., Bers D.M., Grandi E. (2013). Post-Translational Modifications of the Cardiac Na Channel: Contribution of CaMKII-Dependent Phosphorylation to Acquired Arrhythmias. Am. J. Physiol. Heart Circ. Physiol..

[33] Issa Z., Miller J.M., Zipes D.P. (2008). Clinical Arrhythmology and Electrophysiology: A Companion to Braunwald’s Heart Disease: Expert Consult: Online and Print.

[34] Khan M., Aqtash O., Harris D., Costea A., Gerson M. (2022). Ventricular Tachycardia or Fibrillation Storm in Coronavirus Disease. Case Rep. Cardiol..

[35] Shimoda L.A., Polak J. (2011). Hypoxia. 4. Hypoxia and Ion Channel Function. Am. J. Physiol. Cell Physiol..

[36] Afterdepolarization—An Overview|ScienceDirect Topics. https://www.sciencedirect.com/topics/biochemistry-genetics-and-molecular-biology/afterdepolarization.

[37] Nichols C.G., Singh G.K., Grange D.K. (2013). KATP Channels and Cardiovascular Disease. Circ. Res..

[38] Acker H., Bölling B., Delpiano M.A., Dufau E., Görlach A., Holtermann G. (1992). The Meaning of H2O2 Generation in Carotid Body Cells for PO2 Chemoreception. J. Auton. Nerv. Syst..

[39] Maejima Y., Sadoshima J. (2014). SUMOylation. Circ. Res..

[40] Xiong D., Li T., Dai H., Arena A.F., Plant L.D., Goldstein S.A.N. (2017). SUMOylation Determines the Voltage Required to Activate Cardiac IKs Channels. Proc. Natl. Acad. Sci. USA.

[41] Shetty P.M.V., Rangrez A.Y., Frey N. (2020). SUMO Proteins in the Cardiovascular System: Friend or Foe?. J. Biomed. Sci..

[42] Benson M.D., Li Q.-J., Kieckhafer K., Dudek D., Whorton M.R., Sunahara R.K., Iñiguez-Lluhí J.A., Martens J.R. (2007). SUMO Modification Regulates Inactivation of the Voltage-Gated Potassium Channel Kv1.5. Proc. Natl. Acad. Sci. USA.

[43] Rajan S., Plant L.D., Rabin M.L., Butler M.H., Goldstein S.A.N. (2010). Sumoylation Silences the Plasma Membrane Leak K^+^ Channel K2P1. Cell.

[44] van der Heyden M.A., Wijnhoven T.J., Opthof T. (2005). Molecular aspects of adrenergic modulation of cardiac L-type Ca^2+^ channels. Cardiovasc. Res..

[45] Boularan C., Gales C. (2015). Cardiac cAMP: Production, hydrolysis, modulation and detection. Front. Pharmacol..

[46] Stadel J.M., Lefkowitz R.J., Perkins J.P. (1991). The Beta-Adrenergic Receptors.

[47] del Rivero Morfin P.J., Marx S.O., Ben-Johny M., Striessnig J. (2023). Sympathetic Nervous System Regulation of Cardiac Calcium Channels. Voltage-gated Ca^2+^ Channels: Pharmacology, Modulation and their Role in Human Disease. Handbook of Experimental Pharmacology.

[48] Ozawa J., Ohno S., Fujii Y., Makiyama T., Suzuki H., Saitoh A., Horie M. (2018). Differential Diagnosis Between Catecholaminergic Polymorphic Ventricular Tachycardia and Long QT Syndrome Type 1—Modified Schwartz Score—. Circ. J..

[49] Tisdale J.E., Chung M.K., Campbell K.B., Hammadah M., Joglar J.A., Leclerc J., Rajagopalan B., Nursing O., American Heart Association Clinical Pharmacology Committee of the Council on Clinical Cardiology and Council on Cardiovascular and Stroke Nursing (2020). Drug-Induced Arrhythmias: A Scientific Statement From the American Heart Association. Circulation.

[50] Ojo A., McNitt S., Polonsky B., Aktas M.K., Rosero S., Hall B., Kutyifa V., Rao N., Rao N., Goldenberg I. (2024). Digoxin and Risk of Ventricular Tachyarrhythmia and Death in ICD Recipients. JACC Clin. Electrophysiol..

[51] Janousek J., Paul T. (1998). Safety of Oral Propafenone in the Treatment of Arrhythmias in Infants and Children (European Retrospective Multicenter Study). Working Group on Pediatric Arrhythmias and Electrophysiology of the Association of European Pediatric Cardiologists. Am. J. Cardiol..

[52] Li D., Chai S., Wang H., Dong J., Qin C., Du D., Wang Y., Du Q., Liu S. (2023). Drug-Induced QT Prolongation and Torsade de Pointes: A Real-World Pharmacovigilance Study Using the FDA Adverse Event Reporting System Database. Front. Pharmacol..

[53] Haugaa K.H., Bos J.M., Tarrell R.F., Morlan B.W., Caraballo P.J., Ackerman M.J. (2013). Institution-Wide QT Alert System Identifies Patients with a High Risk of Mortality. Mayo Clin. Proc..

[54] Badri M., Patel A., Patel C., Liu G., Goldstein M., Robinson V.M., Xue X., Yang L., Kowey P.R., Yan G.-X. (2015). Mexiletine Prevents Recurrent Torsades de Pointes in Acquired Long QT Syndrome Refractory to Conventional Measures. JACC Clin. Electrophysiol..

[55] Koizumi T., Kamada R., Watanabe M., Yokoshiki H., Temma T., Hagiwara H., Koya T., Nakao M., Kadosaka T., Natsui H. (2022). Predictors of Cardiovascular Mortality after an Electrical Storm in Patients with Structural Heart Disease. J. Cardiol..

[56] Morita H., Zipes D.P., Morita S.T., Lopshire J.C., Wu J. (2009). Epicardial Ablation Eliminates Ventricular Arrhythmias in an Experimental Model of Brugada Syndrome. Heart Rhythm..

[57] Sweeney M.O., Sherfesee L., DeGroot P.J., Wathen M.S., Wilkoff B.L. (2010). Differences in Effects of Electrical Therapy Type for Ventricular Arrhythmias on Mortality in Implantable Cardioverter-Defibrillator Patients. Heart Rhythm..

[58] Roque C., Trevisi N., Silberbauer J., Oloriz T., Mizuno H., Baratto F., Bisceglia C., Sora N., Marzi A., Radinovic A. (2014). Electrical Storm Induced by Cardiac Resynchronization Therapy Is Determined by Pacing on Epicardial Scar and Can Be Successfully Managed by Catheter Ablation. Circ. Arrhythmia Electrophysiol..

[59] Kutyifa V., Zareba W., McNitt S., Singh J., Hall W.J., Polonsky S., Goldenberg I., Huang D.T., Merkely B., Wang P.J. (2013). Left Ventricular Lead Location and the Risk of Ventricular Arrhythmias in the MADIT-CRT Trial. Eur. Heart J..

[60] Özcan E.E., Yılancıoğlu Y., Hasdemir C. (2018). Multipoint Pacing Controlled the Electrical Storm Induced by Cardiac Resynchronization Therapy. EP Eur..

[61] Gonella A., Casile C., Menardi E., Feola M. (2024). Electrical Storm Induced by Cardiac Resynchronization: Efficacy of the Multipoint Pacing Stimulation. Diseases.

[62] Fish J.M., Brugada J., Antzelevitch C. (2005). Potential Proarrhythmic Effects of Biventricular Pacing. J. Am. Coll. Cardiol..

[63] Dusi V., Angelini F., Gravinese C., Frea S., De Ferrari G.M. (2023). Electrical Storm Management in Structural Heart Disease. Eur. Heart J. Suppl..

[64] Kowlgi G.N., Cha Y.-M. (2020). Management of Ventricular Electrical Storm: A Contemporary Appraisal. Europace.

[65] Brugada J., Aguinaga L., Mont L., Betriu A., Mulet J., Sanz G. (2001). Coronary Artery Revascularization in Patients with Sustained Ventricular Arrhythmias in the Chronic Phase of a Myocardial Infarction: Effects on the Electrophysiologic Substrate and Outcome. J. Am. Coll. Cardiol..

[66] Cervantes C.E., Udayappan K.M., Geetha D. (2022). The Devil Is in the Details: Approach to Refractory Hypokalemia. Clevel. Clin. J. Med..

[67] Gorenek B., Wijnmaalen A.P., Goette A., Mert G.O., Porter B., Gustafsson F., Dan G.-A., Ector J., Stuehlinger M., Spartalis M. (2024). Ventricular Arrhythmias in Acute Heart Failure: A Clinical Consensus Statement of the Association for Acute CardioVascular Care, the European Heart Rhythm Association, and the Heart Failure Association of the European Society of Cardiology. Europace.

[68] Tzivoni D., Banai S., Schuger C., Benhorin J., Keren A., Gottlieb S., Stern S. (1988). Treatment of Torsade de Pointes with Magnesium Sulfate. Circulation.

[69] Mandel J.E., Hutchinson M.D., Marchlinski F.E. (2011). Remifentanil-Midazolam Sedation Provides Hemodynamic Stability and Comfort during Epicardial Ablation of Ventricular Tachycardia. J. Cardiovasc. Electrophysiol..

[70] Bristow M.R., Ginsburg R., Umans V., Fowler M., Minobe W., Rasmussen R., Zera P., Menlove R., Shah P., Jamieson S. (1986). Beta 1- and Beta 2-Adrenergic-Receptor Subpopulations in Nonfailing and Failing Human Ventricular Myocardium: Coupling of Both Receptor Subtypes to Muscle Contraction and Selective Beta 1-Receptor down-Regulation in Heart Failure. Circ. Res..

[71] Chatzidou S., Kontogiannis C., Tsilimigras D.I., Georgiopoulos G., Kosmopoulos M., Papadopoulou E., Vasilopoulos G., Rokas S. (2018). Propranolol Versus Metoprolol for Treatment of Electrical Storm in Patients with Implantable Cardioverter-Defibrillator. J. Am. Coll. Cardiol..

[72] Tsagalou E.P., Kanakakis J., Rokas S., Anastasiou-Nana M.I. (2005). Suppression by Propranolol and Amiodarone of an Electrical Storm Refractory to Metoprolol and Amiodarone. Int. J. Cardiol..

[73] Chen P.-S., Doytchinova A. (2018). Why Is Propranolol Better Than Metoprolol in Acute Treatment of Electrical Storm?. J. Am. Coll. Cardiol..

[74] Muser D., Santangeli P., Liang J.J. (2017). Management of Ventricular Tachycardia Storm in Patients with Structural Heart Disease. World J. Cardiol..

[75] Miwa Y., Ikeda T., Mera H., Miyakoshi M., Hoshida K., Yanagisawa R., Ishiguro H., Tsukada T., Abe A., Yusu S. (2010). Effects of Landiolol, an Ultra-Short-Acting Beta1-Selective Blocker, on Electrical Storm Refractory to Class III Antiarrhythmic Drugs. Circ. J..

[76] Dusi V., Angelini F., Gravinese C., Frea S., De Ferrari G.M. (2025). The Role of Antiarrhythmic Drugs and Stellate Ganglion Block in the Acute Management of Electrical Storm. Eur. Heart J. Suppl..

[77] Markman T.M., McBride D.A., Liang J.J. (2018). Catheter Ablation for Ventricular Tachycardia in Patients with Structural Heart Disease. US Cardiol. Rev..

[78] Sapp J.L., Wells G.A., Parkash R., Stevenson W.G., Blier L., Sarrazin J.F., Thibault B., Rivard L., Gula L., Leong-Sit P. (2016). Ventricular Tachycardia Ablation versus Escalation of Antiarrhythmic Drugs. N. Engl. J. Med..

[79] Sorajja D., Munger T.M., Shen W.-K. (2015). Optimal Antiarrhythmic Drug Therapy for Electrical Storm. J. Biomed. Res..

[80] Ho D.S., Zecchin R.P., Richards D.A., Uther J.B., Ross D.L. (1994). Double-Blind Trial of Lignocaine versus Sotalol for Acute Termination of Spontaneous Sustained Ventricular Tachycardia. Lancet.

[81] Yap Y.G., Camm A.J. (2003). Drug Induced QT Prolongation and Torsades de Pointes. Heart.

[82] Charton J., Bouteiller X., Gandjbakhch E., Waintraub X., Klein C., Maury P., Baudinaud P., Marijon E., Tixier R., Baudinet T. (2024). Overdrive Pacing for Ventricular Fibrillation Storm after Myocardial Infarction. Eur. Heart J..

[83] Giannino G., Braia V., Griffith Brookles C., Giacobbe F., D’Ascenzo F., Angelini F., Saglietto A., De Ferrari G.M., Dusi V. (2024). The Intrinsic Cardiac Nervous System: From Pathophysiology to Therapeutic Implications. Biology.

[84] Kingma J.G., Simard D., Rouleau J.R. (2017). Influence of Cardiac Nerve Status on Cardiovascular Regulation and Cardioprotection. World J. Cardiol..

[85] Fukuda K., Kanazawa H., Aizawa Y., Ardell J.L., Shivkumar K. (2015). Cardiac Innervation and Sudden Cardiac Death. Circ. Res..

[86] Do D.H., Bradfield J., Ajijola O.A., Vaseghi M., Le J., Rahman S., Mahajan A., Nogami A., Boyle N.G., Shivkumar K. (2017). Thoracic Epidural Anesthesia Can Be Effective for the Short-Term Management of Ventricular Tachycardia Storm. J. Am. Heart Assoc..

[87] Basantwani S., Shinde S.R., Tendolkar B. (2019). Management of Ventricular Storm with Thoracic Epidural Anesthesia. Ann. Card. Anaesth..

[88] Tian Y., Wittwer E.D., Kapa S., McLeod C.J., Xiao P., Noseworthy P.A., Mulpuru S.K., Deshmukh A.J., Lee H.-C., Ackerman M.J. (2019). Effective Use of Percutaneous Stellate Ganglion Blockade in Patients with Electrical Storm. Circ. Arrhythmia Electrophysiol..

[89] Sanghai S., Abbott N.J., Dewland T.A., Henrikson C.A., Elman M.R., Wollenberg M., Ivie R., Gonzalez-Sotomayor J., Nazer B. (2021). Stellate Ganglion Blockade with Continuous Infusion Versus Single Injection for Treatment of Ventricular Arrhythmia Storm. JACC Clin. Electrophysiol..

[90] Benali K., Ninni S., Guenancia C., Mohammed R., Decaudin D., Bourdrel O., Salaun A., Yvorel C., Groussin P., Pavin D. (2024). Impact of Catheter Ablation of Electrical Storm on Survival: A Propensity Score-Matched Analysis. Clin. Electrophysiol..

[91] Khan U., Khlidj Y., Ibrahim A.A., Amin A.M., Rakab M.S., AlBarakat M.M., Khan M.H., Majeed Z., Imran M., Ali J. (2025). Catheter Ablation versus Medical Therapy for Ventricular Tachycardia in Patients with Ischemic Heart Disease: A Systematic Review and Meta-Analysis of Randomized Controlled Trials. Indian Pacing Electrophysiol. J..

[92] Deyell M.W., Doucette S., Parkash R., Nault I., Gula L., Gray C., Gardner M., Sterns L.D., Healey J.S., Essebag V. (2022). Ventricular Tachycardia Characteristics and Outcomes with Catheter Ablation vs. Antiarrhythmic Therapy: Insights from the VANISH Trial. Europace.

[93] Sapp J.L., Tang A.S.L., Parkash R., Stevenson W.G., Healey J.S., Wells G. (2024). A Randomized Clinical Trial of Catheter Ablation and Antiarrhythmic Drug Therapy for Suppression of Ventricular Tachycardia in Ischemic Cardiomyopathy: The VANISH2 Trial. Am. Heart J..

[94] Tilz R.R., Kuck K.H., Kääb S., Wegscheider K., Thiem A., Wenzel B., Willems S., Steven D. (2019). Rationale and Design of BERLIN VT Study: A Multicenter Randomised Trial Comparing Preventive versus Deferred Ablation of Ventricular Tachycardia. BMJ Open.

[95] Kumar R., Amadio J.M., Luk A.C., Bhaskaran A., Ha A.C.T. (2025). Extracorporeal Membrane Oxygenation for Patients with Electrical Storm or Refractory Ventricular Arrhythmias: Management and Outcomes. Can. J. Cardiol..

[96] Mariani M.V., Pierucci N., Cipollone P., Vignaroli W., Piro A., Compagnucci P., Matteucci A., Chimenti C., Pandozi C., Dello Russo A. (2024). Mechanical Circulatory Support Systems in the Management of Ventricular Arrhythmias: A Contemporary Overview. J. Clin. Med..

[97] Dyer S., Mogni B., Gottlieb M. (2020). Electrical Storm: A Focused Review for the Emergency Physician. Am. J. Emerg. Med..

[98] Streitner F., Kuschyk J., Veltmann C., Mahl E., Dietrich C., Schimpf R., Doesch C., Streitner I., Wolpert C., Borggrefe M. (2011). Predictors of Electrical Storm Recurrences in Patients with Implantable Cardioverter-Defibrillators. Europace.

[99] Arya A., Haghjoo M., Dehghani M.R., Fazelifar A.F., Nikoo M.-H., Bagherzadeh A., Sadr-Ameli M.A. (2006). Prevalence and Predictors of Electrical Storm in Patients with Implantable Cardioverter-Defibrillator. Am. J. Cardiol..

[100] Zaugg C.E., Wu S.T., Barbosa V., Buser P.T., Wikman-Coffelt J., Parmley W.W., Lee R.J. (1998). Ventricular Fibrillation-Induced Intracellular Ca^2+^ Overload Causes Failed Electrical Defibrillation and Post-Shock Reinitiation of Fibrillation. J. Mol. Cell Cardiol..

[101] Jung W., Manz M., Pizzulli L., Pfeiffer D., Lüderitz B. (1992). Effects of Chronic Amiodarone Therapy on Defibrillation Threshold. Am. J. Cardiol..

[102] Mehta V.S., Elliott M.K., Sidhu B.S., Gould J., Porter B., Niederer S., Rinaldi C.A. (2021). Multipoint Pacing for Cardiac Resynchronisation Therapy in Patients with Heart Failure: A Systematic Review and Meta-Analysis. J. Cardiovasc. Electrophysiol..

[103] Rinaldi C.A., Burri H., Thibault B., Curnis A., Rao A., Gras D., Sperzel J., Singh J.P., Biffi M., Bordachar P. (2015). A Review of Multisite Pacing to Achieve Cardiac Resynchronization Therapy. Europace.

[104] Pujol-Lopez M., Jiménez-Arjona R., Garre P., Guasch E., Borràs R., Doltra A., Ferró E., García-Ribas C., Niebla M., Carro E. (2022). Conduction System Pacing vs Biventricular Pacing in Heart Failure and Wide QRS Patients: LEVEL-AT Trial. JACC Clin. Electrophysiol..

[105] Kim J.A., Kim S.E., Ellenbogen K.A., Vijayaraman P., Chelu M.G. (2023). Clinical Outcomes of Conduction System Pacing versus Biventricular Pacing for Cardiac Resynchronization Therapy: A Systematic Review and Meta-Analysis. J. Cardiovasc. Electrophysiol..

[106] Cleland J.G.F., Daubert J.-C., Erdmann E., Freemantle N., Gras D., Kappenberger L., Tavazzi L. (2005). Cardiac Resynchronization-Heart Failure (CARE-HF) Study Investigators The Effect of Cardiac Resynchronization on Morbidity and Mortality in Heart Failure. N. Engl. J. Med..

[107] Abraham W.T., Fisher W.G., Smith A.L., Delurgio D.B., Leon A.R., Loh E., Kocovic D.Z., Packer M., Clavell A.L., Hayes D.L. (2002). Cardiac Resynchronization in Chronic Heart Failure. N. Engl. J. Med..

[108] Ploux S., Strik M., van Hunnik A., van Middendorp L., Kuiper M., Prinzen F.W. (2014). Acute Electrical and Hemodynamic Effects of Multisite Left Ventricular Pacing for Cardiac Resynchronization Therapy in the Dyssynchronous Canine Heart. Heart Rhythm..

[109] Herweg B., Sharma P.S., Cano Ó., Ponnusamy S.S., Zanon F., Jastrzebski M., Zou J., Chelu M.G., Vernooy K., Whinnett Z.I. (2024). Arrhythmic Risk in Biventricular Pacing Compared with Left Bundle Branch Area Pacing: Results From the I-CLAS Study. Circulation.

[110] Ninni S., Gallot-Lavallée T., Klein C., Longère B., Brigadeau F., Potelle C., Crop F., Rault E., Decoene C., Lacornerie T. (2022). Stereotactic Radioablation for Ventricular Tachycardia in the Setting of Electrical Storm. Circ. Arrhythmia Electrophysiol..

[111] Najjar E., Dalén M., Schwieler J., Lund L.H. (2021). A Case Report about Successful Treatment of Refractory Ventricular Tachycardia with Ablation under Prolonged Haemodynamic Support with Extracorporeal Membrane Oxygenation. Eur. Heart J. Case Rep..

[112] Sertic F., Bermudez C., Rame J.E. (2020). Venoarterial Extracorporeal Membrane Oxygenation as a Bridge to Recovery or Bridge to Heart Replacement Therapy in Refractory Cardiogenic Shock. Curr. Heart Fail. Rep..

[113] Khiabani A.J., Pawale A. (2024). Extracorporeal Membrane Oxygenation in Cardiogenic Shock: Execution Is Something; Timing Is Everything?. J. Am. Heart Assoc..

[114] Eckman P.M., Katz J.N., El Banayosy A., Bohula E.A., Sun B., van Diepen S. (2019). Veno-Arterial Extracorporeal Membrane Oxygenation for Cardiogenic Shock. Circulation.

[115] Choi K.H., Yang J.H., Hong D., Park T.K., Lee J.M., Song Y.B., Hahn J.-Y., Choi S.-H., Choi J.-H., Chung S.R. (2020). Optimal Timing of Venoarterial-Extracorporeal Membrane Oxygenation in Acute Myocardial Infarction Patients Suffering From Refractory Cardiogenic Shock. Circ. J..

[116] Rajakumar H.K. (2024). Enhancing the Clinical Application of VA-ECMO Timing in Cardiac Surgery for Improved Patient Outcomes. Cardiothorac. Surg..

[117] Schwartz P.J., Stramba-Badiale M., Crotti L., Pedrazzini M., Besana A., Bosi G., Gabbarini F., Goulene K., Insolia R., Mannarino S. (2009). Prevalence of the Congenital Long-QT Syndrome. Circulation.

[118] Nakano Y., Shimizu W. (2016). Genetics of Long-QT Syndrome. J. Hum. Genet..

[119] Postema P.G., van Eck H.J.R., Opthof T., van Herpen G., van Dessel P.F.H.M., Priori S.G., Wolpert C., Borggrefe M., Kors J.A., Wilde A.A.M. (2009). IK1 Modulates the U-Wave: Insights in a 100-Year-Old Enigma. Heart Rhythm..

[120] Schwartz P.J., Crotti L. (2011). QTc Behavior During Exercise and Genetic Testing for the Long-QT Syndrome. Circulation.

[121] Porta-Sánchez A., Spillane D.R., Harris L., Xue J., Dorsey P., Care M., Chauhan V., Gollob M.H., Spears D.A. (2017). T-Wave Morphology Analysis in Congenital Long QT Syndrome Discriminates Patients From Healthy Individuals. JACC Clin. Electrophysiol..

[122] Han W., Wang Z., Nattel S. (2001). Slow Delayed Rectifier Current and Repolarization in Canine Cardiac Purkinje Cells. Am. J. Physiol. Heart Circ. Physiol..

[123] MacIntyre C.J., Ackerman M.J. (2023). Personalized Care in Long QT Syndrome: Better Management, More Sports, and Fewer Devices. Card. Electrophysiol. Clin..

[124] Heidbuchel H., Arbelo E., D’Ascenzi F., Borjesson M., Boveda S., Castelletti S., Miljoen H., Mont L., Niebauer J., Papadakis M. (2021). Recommendations for Participation in Leisure-Time Physical Activity and Competitive Sports of Patients with Arrhythmias and Potentially Arrhythmogenic Conditions. Part 2: Ventricular Arrhythmias, Channelopathies, and Implantable Defibrillators. Europace.

[125] Lampert R., Day S., Ainsworth B., Burg M., Marino B.S., Salberg L., Esteban M.T.T., Abrams D.J., Aziz P.F., Barth C. (2024). Vigorous Exercise in Patients with Congenital Long QT Syndrome: Results of the Prospective, Observational, Multinational LIVE-LQTS Study. Circulation.

[126] Wilde A.A.M., Amin A.S., Postema P.G. (2022). Diagnosis, Management and Therapeutic Strategies for Congenital Long QT Syndrome. Heart.

[127] Pérez-Riera A.R., Barbosa-Barros R., Daminello Raimundo R., da Costa de Rezende Barbosa M.P., Esposito Sorpreso I.C., de Abreu L.C. (2017). The Congenital Long QT Syndrome Type 3: An Update. Indian Pacing Electrophysiol. J..

[128] Clancy C.E., Tateyama M., Kass R.S. (2002). Insights into the Molecular Mechanisms of Bradycardia-Triggered Arrhythmias in Long QT-3 Syndrome. J. Clin. Investig..

[129] Qi M., Ma S., Liu J., Liu X., Wei J., Lu W.-J., Zhang S., Chang Y., Zhang Y., Zhong K. (2024). In Vivo Base Editing of Scn5a Rescues Type 3 Long QT Syndrome in Mice. Circulation.

[130] Mazzanti A., Maragna R., Vacanti G., Monteforte N., Bloise R., Marino M., Braghieri L., Gambelli P., Memmi M., Pagan E. (2018). Interplay Between Genetic Substrate, QTc Duration, and Arrhythmia Risk in Patients with Long QT Syndrome. J. Am. Coll. Cardiol..

[131] Mazzanti A., Trancuccio A., Kukavica D., Pagan E., Wang M., Mohsin M., Peterson D., Bagnardi V., Zareba W., Priori S.G. (2022). Independent Validation and Clinical Implications of the Risk Prediction Model for Long QT Syndrome (1-2-3-LQTS-Risk). Europace.

[132] Priori S.G., Napolitano C., Schwartz P.J., Grillo M., Bloise R., Ronchetti E., Moncalvo C., Tulipani C., Veia A., Bottelli G. (2004). Association of Long QT Syndrome Loci and Cardiac Events Among Patients Treated with β-Blockers. JAMA.

[133] Alhourani N., Wolfes J., Könemann H., Ellermann C., Frommeyer G., Güner F., Lange P.S., Reinke F., Köbe J., Eckardt L. (2024). Relevance of Mexiletine in the Era of Evolving Antiarrhythmic Therapy of Ventricular Arrhythmias. Clin. Res. Cardiol..

[134] Crotti L., Neves R., Dagradi F., Musu G., Giannetti F., Bos J.M., Barbieri M., Cerea P., Giovenzana F.L.F., Torchio M. (2024). Therapeutic Efficacy of Mexiletine for Long QT Syndrome Type 2: Evidence From Human Induced Pluripotent Stem Cell-Derived Cardiomyocytes, Transgenic Rabbits, and Patients. Circulation.

[135] Antzelevitch C., Burashnikov A., Sicouri S., Belardinelli L. (2011). Electrophysiological Basis for the Antiarrhythmic Actions of Ranolazine. Heart Rhythm..

[136] Davies R.A., Ladouceur V.B., Green M.S., Joza J., Juurlink D.N., Krahn A.D., McMurtry M.S., Roberts J.D., Roston T.M., Sanatani S. (2023). The 2023 Canadian Cardiovascular Society Clinical Practice Update on Management of the Patient with a Prolonged QT Interval. Can. J. Cardiol..

[137] Horner J.M., Kinoshita M., Webster T.L., Haglund C.M., Friedman P.A., Ackerman M.J. (2010). Implantable Cardioverter Defibrillator Therapy for Congenital Long QT Syndrome: A Single-Center Experience. Heart Rhythm..

[138] Olde Nordkamp L.R.A., Postema P.G., Knops R.E., van Dijk N., Limpens J., Wilde A.A.M., de Groot J.R. (2016). Implantable Cardioverter-Defibrillator Harm in Young Patients with Inherited Arrhythmia Syndromes: A Systematic Review and Meta-Analysis of Inappropriate Shocks and Complications. Heart Rhythm..

[139] Schneider L., Begovic M., Zhou X., Hamdani N., Akin I., El-Battrawy I. (2025). Catecholaminergic Polymorphic Ventricular Tachycardia: Advancing From Molecular Insights to Preclinical Models. J. Am. Heart Assoc..

[140] Priori S.G., Napolitano C., Tiso N., Memmi M., Vignati G., Bloise R., Sorrentino V., Danieli G.A.A. (2001). Mutations in the cardiac ryanodine receptor gene (hRyR2) underlie catecholaminergic polymorphic ventricular tachycardia. Circulation.

[141] Novak A., Barad L., Zeevi-Levin N., Shick R., Shtrichman R., Lorber A., Itskovitz-Eldor J., Binah O. (2012). Cardiomyocytes Generated from CPVTD307H Patients Are Arrhythmogenic in Response to β-Adrenergic Stimulation. J. Cell Mol. Med..

[142] Zhao Y.-T., Valdivia C.R., Gurrola G.B., Powers P.P., Willis B.C., Moss R.L., Jalife J., Valdivia H.H. (2015). Arrhythmogenesis in a Catecholaminergic Polymorphic Ventricular Tachycardia Mutation That Depresses Ryanodine Receptor Function. Proc. Natl. Acad. Sci. USA.

[143] Priori S.G., Chen S.W. (2011). Inherited Dysfunction of Sarcoplasmic Reticulum Ca^2+^ Handling and Arrhythmogenesis. Circ. Res..

[144] Leenhardt A., Denjoy I., Guicheney P. (2012). Catecholaminergic Polymorphic Ventricular Tachycardia. Circ. Arrhythmia Electrophysiol..

[145] Viatchenko-Karpinski S., Terentyev D., Györke I., Terentyeva R., Volpe P., Priori S.G., Napolitano C., Nori A., Williams S.C., Györke S. (2004). Abnormal Calcium Signaling and Sudden Cardiac Death Associated with Mutation of Calsequestrin. Circ. Res..

[146] Sun B., Yao J., Ni M., Wei J., Zhong X., Guo W., Zhang L., Wang R., Belke D., Chen Y.-X. (2021). Cardiac Ryanodine Receptor Calcium Release Deficiency Syndrome. Sci. Transl. Med..

[147] Li Y., Wei J., Guo W., Sun B., Estillore J.P., Wang R., Yoruk A., Roston T.M., Sanatani S., Wilde A.A.M. (2021). Human RyR2 (Ryanodine Receptor 2) Loss-of-Function Mutations. Circ. Arrhythmia Electrophysiol..

[148] DIAGNOSE CRDS—Research Studies—PHRI—Population Health Research Institute of Canada. https://www.phri.ca/research/diagnose-crds/.

[149] Ni M., Dadon Z., Ormerod J.O.M., Saenen J., Hoeksema W.F., Antiperovitch P., Tadros R., Christiansen M.K., Steinberg C., Arnaud M. (2024). A Clinical Diagnostic Test for Calcium Release Deficiency Syndrome. JAMA.

[150] Kallas D., Roberts J.D., Sanatani S., Roston T.M. (2023). Calcium Release Deficiency Syndrome: A New Inherited Arrhythmia Syndrome. Card. Electrophysiol. Clin..

[151] Bellamy D., Nuthall G., Dalziel S., Skinner J.R. (2019). Catecholaminergic Polymorphic Ventricular Tachycardia: The Cardiac Arrest Where Epinephrine Is Contraindicated. Pediatr. Crit. Care Med..

[152] Marjamaa A., Hiippala A., Arrhenius B., Lahtinen A.M., Kontula K., Toivonen L., Happonen J.-M., Swan H. (2012). Intravenous Epinephrine Infusion Test in Diagnosis of Catecholaminergic Polymorphic Ventricular Tachycardia. J. Cardiovasc. Electrophysiol..

[153] Fagundes A., DE Magalhaes L.P., Russo M., Xavier E. (2010). Pharmacological Treatment of Electrical Storm in Cathecolaminergic Polymorphic Ventricular Tachycardia. Pacing Clin. Electrophysiol..

[154] Aggarwal A., Stolear A., Alam M.M., Vardhan S., Dulgher M., Jang S.-J., Zarich S.W. (2024). Catecholaminergic Polymorphic Ventricular Tachycardia: Clinical Characteristics, Diagnostic Evaluation and Therapeutic Strategies. J. Clin. Med..

[155] Kaneshiro T., Naruse Y., Nogami A., Tada H., Yoshida K., Sekiguchi Y., Murakoshi N., Kato Y., Horigome H., Kawamura M. (2012). Successful Catheter Ablation of Bidirectional Ventricular Premature Contractions Triggering Ventricular Fibrillation in Catecholaminergic Polymorphic Ventricular Tachycardia with RyR2 Mutation. Circ. Arrhythmia Electrophysiol..

[156] Shen L., Liu S., Hu F., Zhang Z., Li J., Lai Z., Zheng L., Yao Y. (2023). Electrophysiological Characteristics and Ablation Outcomes in Patients with Catecholaminergic Polymorphic Ventricular Tachycardia. J. Am. Heart Assoc..

[157] Brugada R., Campuzano O., Sarquella-Brugada G., Brugada J., Brugada P. (2014). Brugada Syndrome. Methodist. Debakey Cardiovasc. J..

[158] Bayés de Luna A., Brugada J., Baranchuk A., Borggrefe M., Breithardt G., Goldwasser D., Lambiase P., Riera A.P., Garcia-Niebla J., Pastore C. (2012). Current Electrocardiographic Criteria for Diagnosis of Brugada Pattern: A Consensus Report. J. Electrocardiol..

[159] Antzelevitch C., Brugada P., Borggrefe M., Brugada J., Brugada R., Corrado D., Gussak I., LeMarec H., Nademanee K., Perez Riera A.R. (2005). Brugada Syndrome: Report of the Second Consensus Conference. Circulation.

[160] Shelke A., Tachil A., Saggu D., Jesuraj M.L., Yalagudri S., Narasimhan C. (2018). Catheter Ablation for Electrical Storm in Brugada Syndrome: Results of Substrate Based Ablation. Indian Heart J..

[161] El-Battrawy I., Roterberg G., Kowitz J., Aweimer A., Lang S., Mügge A., Zhou X., Akin I. (2022). Incidence, Recurrence and Management of Electrical Storm in Brugada Syndrome. Front. Cardiovasc. Med..

[162] Chakraborty P., Rahimi M., Suszko A.M., Massin S., Laksman Z., Spears D., Gollob M.H., Chauhan V.S. (2024). Exercise-Induced QRS Prolongation in Brugada Syndrome. JACC Clin. Electrophysiol..

[163] Postema P.G., Wolpert C., Amin A.S., Probst V., Borggrefe M., Roden D.M., Priori S.G., Tan H.L., Hiraoka M., Brugada J. (2009). Drugs and Brugada Syndrome Patients: Review of the Literature, Recommendations, and an up-to-Date Website (Www.Brugadadrugs.Org). Heart Rhythm..

[164] Postema P.G., Neville J., de Jong J.S.S.G., Romero K., Wilde A.A.M., Woosley R.L. (2013). Safe Drug Use in Long QT Syndrome and Brugada Syndrome: Comparison of Website Statistics. Europace.

[165] Chockalingam P., Clur S.-A.B., Breur J.M.P.J., Kriebel T., Paul T., Rammeloo L.A., Wilde A.A.M., Blom N.A. (2012). The Diagnostic and Therapeutic Aspects of Loss-of-Function Cardiac Sodium Channelopathies in Children. Heart Rhythm..

[166] Verberne H.J., Blom M.T., Bardai A., Karemaker J.M., Tan H.L. (2022). An Inherited Sudden Cardiac Arrest Syndrome May Be Based on Primary Myocardial and Autonomic Nervous System Abnormalities. Heart Rhythm..

[167] Rivera-Juárez A., Hernández-Romero I., Puertas C., Zhang-Wang S., Sánchez-Álamo B., Martins R., Figuera C., Guillem M.S., Climent A.M., Fernández-Avilés F. (2019). Clinical Characteristics and Electrophysiological Mechanisms Underlying Brugada ECG in Patients with Severe Hyperkalemia. J. Am. Heart Assoc..

[168] Teraoka J.T., Tang J.J., Noubiap J.J., Dewland T.A., Marcus G.M. (2023). Abstract 17249: Epidemiology of Wolff-Parkinson-White Syndrome Among Acute Care Recipients in California. Circulation.

[169] Page R.L., Joglar J.A., Caldwell M.A., Calkins H., Conti J.B., Deal B.J., Estes N.A.M., Field M.E., Goldberger Z.D., Hammill S.C. (2016). 2015 ACC/AHA/HRS Guideline for the Management of Adult Patients with Supraventricular Tachycardia: A Report of the American College of Cardiology/American Heart Association Task Force on Clinical Practice Guidelines and the Heart Rhythm Society. J. Am. Coll. Cardiol..

[170] Vătășescu R.G., Paja C.S., Șuș I., Cainap S., Moisa Ș.M., Cinteză E.E. (2024). Wolf–Parkinson–White Syndrome: Diagnosis, Risk Assessment, and Therapy—An Update. Diagnostics.

[171] Waller B.F., Gering L.E., Branyas N.A., Slack J.D. (1993). Anatomy, Histology, and Pathology of the Cardiac Conduction System—Part IV. Clin. Cardiol..

[172] Chrispin J., Calkins H., Camm A.J., Lüscher T.F., Maurer G., Serruys P.W. (2018). Accessory Pathways-Related Tachycardias: Wolff–Parkinson–White Syndrome and Atrioventricular Reentrant Tachycardias. The ESC Textbook of Cardiovascular Medicine.

[173] Milstein S., Sharma A.D., Klein G.J. (1986). Electrophysiologic Profile of Asymptomatic Wolff-Parkinson-White Pattern. Am. J. Cardiol..

[174] Obeyesekere M., Gula L.J., Skanes A.C., Leong-Sit P., Klein G.J. (2012). Risk of Sudden Death in Wolff-Parkinson-White Syndrome. Circulation.

[175] Centurion O.A. (2011). Atrial Fibrillation in the Wolff-Parkinson-White Syndrome. J. Atr. Fibrillation.

[176] Haissaguerre M., Fischer B., Labbé T., Lemétayer P., Montserrat P., d’Ivernois C., Dartigues J.F., Warin J.F. (1992). Frequency of Recurrent Atrial Fibrillation after Catheter Ablation of Overt Accessory Pathways. Am. J. Cardiol..

[177] 2012 HRS EHRA ECAS AF Ablation—Full Length Version|Heart Rhythm Society. https://www.hrsonline.org/resource/2012-hrsehraecas-expert-consensus-statement-catheter-and-surgical-ablation-atrial-fibrillation/.

[178] Fujimura O., Klein G.J., Yee R., Sharma A.D. (1990). Mode of Onset of Atrial Fibrillation in the Wolff-Parkinson-White Syndrome: How Important Is the Accessory Pathway?. J. Am. Coll. Cardiol..

[179] Santinelli V., Radinovic A., Manguso F., Vicedomini G., Gulletta S., Paglino G., Mazzone P., Ciconte G., Sacchi S., Sala S. (2009). The Natural History of Asymptomatic Ventricular Pre-Excitation: A Long-Term Prospective Follow-Up Study of 184 Asymptomatic Children. J. Am. Coll. Cardiol..

[180] Di Mambro C., Russo M.S., Righi D., Placidi S., Palmieri R., Silvetti M.S., Gimigliano F., Prosperi M., Drago F. (2015). Ventricular Pre-Excitation: Symptomatic and Asymptomatic Children Have the Same Potential Risk of Sudden Cardiac Death. Europace.

[181] Jemtrén A., Saygi S., Åkerström F., Asaad F., Bourke T., Braunschweig F., Carnlöf C., Drca N., Insulander P., Kennebäck G. (2024). Risk Assessment in Patients with Symptomatic and Asymptomatic Pre-Excitation. EP Eur..

[182] Ponti R.D., Marazzato J., Marazzi R., Doni L.A., Salerno-Uriarte J.A. (2018). Pre-eccitazione ventricolare in assenza di sintomi: Che strada percorrere. G. Ital. Di Cardiol..

[183] Escudero C.A., Ceresnak S.R., Collins K.K., Pass R.H., Aziz P.F., Blaufox A.D., Ortega M.C., Cannon B.C., Cohen M.I., Dechert B.E. (2020). Loss of Ventricular Preexcitation during Noninvasive Testing Does Not Exclude High-Risk Accessory Pathways: A Multicenter Study of WPW in Children. Heart Rhythm..

[184] Turley A.J., Murray S., Thambyrajah J. (2008). Pre-Excited Atrial Fibrillation Triggered by Intravenous Adenosine: A Commonly Used Drug with Potentially Life-Threatening Adverse Effects. Emerg. Med. J..

[185] Kaakeh Y., Overholser B.R., Lopshire J.C., Tisdale J.E. (2012). Drug-Induced Atrial Fibrillation. Drugs.

[186] Ibrahim Ali Sherdia A.F., Abdelaal S.A., Hasan M.T., Elsayed E., Mare’y M., Nawar A.A., Abdelsalam A., Abdelgader M.Z., Adam A., Abozaid M. (2023). The Success Rate of Radiofrequency Catheter Ablation in Wolff-Parkinson-White-Syndrome Patients: A Systematic Review and Meta-Analysis. Indian Heart J..

[187] Burke B.J., Assaad I.E., Liu W., Kanj M., Wazni O.M., Callahan T.D., Baranowski B., Saarel E.V., Heilbronner A., Aziz P.F. (2024). Underestimated Recurrence Rates after Ablation for Wolff-Parkinson-White Syndrome and Impact on Follow-up Practices. Heart Rhythm..

[188] Antzelevitch C., Yan G.-X. (2011). J-Wave Syndromes. from Cell to Bedside. J. Electrocardiol..

[189] Kaneko Y., Horie M., Niwano S., Kusano K.F., Takatsuki S., Kurita T., Mitsuhashi T., Nakajima T., Irie T., Hasegawa K. (2014). Electrical Storm in Patients with Brugada Syndrome Is Associated with Early Repolarization. Circ. Arrhythmia Electrophysiol..

[190] Rosso R., Kogan E., Belhassen B., Rozovski U., Scheinman M.M., Zeltser D., Halkin A., Steinvil A., Heller K., Glikson M. (2008). J-Point Elevation in Survivors of Primary Ventricular Fibrillation and Matched Control Subjects: Incidence and Clinical Significance. J. Am. Coll. Cardiol..

[191] Antzelevitch C., Yan G.-X., Ackerman M.J., Borggrefe M., Corrado D., Guo J., Gussak I., Hasdemir C., Horie M., Huikuri H. (2016). J-Wave Syndromes Expert Consensus Conference Report: Emerging Concepts and Gaps in Knowledge. Heart Rhythm..

[192] Early Repolarization Syndrome—Cardiovascular Disorders. https://www.msdmanuals.com/professional/cardiovascular-disorders/arrhythmogenic-cardiac-disorders/early-repolarization-syndrome.

[193] Haïssaguerre M., Derval N., Sacher F., Jesel L., Deisenhofer I., de Roy L., Pasquié J.-L., Nogami A., Babuty D., Yli-Mayry S. (2008). Sudden Cardiac Arrest Associated with Early Repolarization. N. Engl. J. Med..

[194] Chen X., Barajas-Martínez H., Xia H., Zhang Z., Chen G., Yang B., Jiang H., Antzelevitch C., Hu D. (2021). Clinical and Functional Genetic Characterization of the Role of Cardiac Calcium Channel Variants in the Early Repolarization Syndrome. Front. Cardiovasc. Med..

[195] Nademanee K., Haissaguerre M., Hocini M., Nogami A., Cheniti G., Duchateau J., Behr E.R., Saba M., Bokan R., Lou Q. (2019). Mapping and Ablation of Ventricular Fibrillation Associated with Early Repolarization Syndrome. Circulation.

[196] Cheniti G., Vlachos K., Meo M., Puyo S., Thompson N., Denis A., Duchateau J., Takigawa M., Martin C., Frontera A. (2018). Mapping and Ablation of Idiopathic Ventricular Fibrillation. Front. Cardiovasc. Med..

[197] Gussak I., Brugada P., Brugada J., Wright R.S., Kopecky S.L., Chaitman B.R., Bjerregaard P. (2000). Idiopathic Short QT Interval: A New Clinical Syndrome?. Cardiology.

[198] Cordeiro J.M., Brugada R., Wu Y.S., Hong K., Dumaine R. (2005). Modulation of I(Kr) Inactivation by Mutation N588K in KCNH2: A Link to Arrhythmogenesis in Short QT Syndrome. Cardiovasc. Res..

[199] Wu X., Larsson H.P. (2020). Insights into Cardiac IKs (KCNQ1/KCNE1) Channels Regulation. Int. J. Mol. Sci..

[200] Priori S.G., Pandit S.V., Rivolta I., Berenfeld O., Ronchetti E., Dhamoon A., Napolitano C., Anumonwo J., di Barletta M.R., Gudapakkam S. (2005). A Novel Form of Short QT Syndrome (SQT3) Is Caused by a Mutation in the KCNJ2 Gene. Circ. Res..

[201] Li Y., Wang K., Li Q., Hancox J.C., Zhang H., Saucerman J.J. (2021). Reciprocal Interaction between IK1 and If in Biological Pacemakers: A Simulation Study. PLOS Comput. Biol..

[202] The Short QT Syndrome | SpringerLink. https://link.springer.com/chapter/10.1007/978-3-031-33588-4_26.

[203] Sun Y., Timofeyev V., Dennis A., Bektik E., Wan X., Laurita K.R., Deschênes I., Li R.A., Fu J.-D. (2017). A Singular Role of IK1 Promoting the Development of Cardiac Automaticity during Cardiomyocyte Differentiation by IK1 -Induced Activation of Pacemaker Current. Stem Cell Rev. Rep..

[204] Gaita F., Giustetto C., Bianchi F., Wolpert C., Schimpf R., Riccardi R., Grossi S., Richiardi E., Borggrefe M. (2003). Short QT syndrome: A familial cause of sudden death. Circulation.

[205] Brugada J., Katritsis D.G., Arbelo E., Arribas F., Bax J.J., Blomström-Lundqvist C., Calkins H., Corrado D., Deftereos S.G., Diller G.-P. (2020). 2019 ESC Guidelines for the Management of Patients with Supraventricular tachycardiaThe Task Force for the Management of Patients with Supraventricular Tachycardia of the European Society of Cardiology (ESC). Eur. Heart J..

[206] Al-Khatib S.M., Stevenson W.G., Ackerman M.J., Bryant W.J., Callans D.J., Curtis A.B., Deal B.J., Dickfeld T., Field M.E., Fonarow G.C. (2018). 2017 AHA/ACC/HRS Guideline for Management of Patients with Ventricular Arrhythmias and the Prevention of Sudden Cardiac Death. Circulation.

[207] Gaita F., Giustetto C., Bianchi F., Schimpf R., Haissaguerre M., Calò L., Brugada R., Antzelevitch C., Borggrefe M., Wolpert C. (2004). Short QT Syndrome: Pharmacological Treatment. J. Am. Coll. Cardiol..

[208] Bun S.-S., Maury P., Giustetto C., Deharo J.-C. (2012). Electrical Storm in Short-QT Syndrome Successfully Treated with Isoproterenol. J. Cardiovasc. Electrophysiol..

[209] Towbin J.A., McKenna W.J., Abrams D.J., Ackerman M.J., Calkins H., Darrieux F.C.C., Daubert J.P., de Chillou C., DePasquale E.C., Desai M.Y. (2019). 2019 HRS Expert Consensus Statement on Evaluation, Risk Stratification, and Management of Arrhythmogenic Cardiomyopathy. Heart Rhythm..

[210] Stanciulescu L.A., Dorobantu M., Vatasescu R. (2025). Targeting Ventricular Arrhythmias in Non-Ischemic Patients: Advances in Diagnosis and Treatment. Diagnostics.

[211] Corrado D., Basso C., Thiene G., McKenna W.J., Davies M.J., Fontaliran F., Nava A., Silvestri F., Blomstrom-Lundqvist C., Wlodarska E.K. (1997). Spectrum of Clinicopathologic Manifestations of Arrhythmogenic Right Ventricular Cardiomyopathy/Dysplasia: A Multicenter Study. J. Am. Coll. Cardiol..

[212] Corrado D., Wichter T., Link M.S., Hauer R.N.W., Marchlinski F.E., Anastasakis A., Bauce B., Basso C., Brunckhorst C., Tsatsopoulou A. (2015). Treatment of Arrhythmogenic Right Ventricular Cardiomyopathy/Dysplasia. Circulation.

[213] Calkins H., Corrado D., Marcus F. (2017). Risk Stratification in Arrhythmogenic Right Ventricular Cardiomyopathy. Circulation.

[214] Wichter T., Borggrefe M., Haverkamp W., Chen X., Breithardt G. (1992). Efficacy of Antiarrhythmic Drugs in Patients with Arrhythmogenic Right Ventricular Disease. Results in Patients with Inducible and Noninducible Ventricular Tachycardia. Circulation.

[215] Marcus G.M., Glidden D.V., Polonsky B., Zareba W., Smith L.M., Cannom D.S., Estes N.A.M., Marcus F., Scheinman M.M. (2009). Multidisciplinary Study of Right Ventricular Dysplasia Investigators Efficacy of Antiarrhythmic Drugs in Arrhythmogenic Right Ventricular Cardiomyopathy: A Report from the North American ARVC Registry. J. Am. Coll. Cardiol..

[216] Shaikh T., Nguyen D., Dugal J.K., DiCaro M.V., Yee B., Houshmand N., Lei K., Namazi A. (2025). Arrhythmogenic Right Ventricular Cardiomyopathy: A Comprehensive Review. J. Cardiovasc. Dev. Dis..

[217] Daimee U.A., Assis F.R., Murray B., Tichnell C., James C.A., Calkins H., Tandri H. (2021). Clinical Outcomes of Catheter Ablation of Ventricular Tachycardia in Patients with Arrhythmogenic Right Ventricular Cardiomyopathy: Insights from the Johns Hopkins ARVC Program. Heart Rhythm..

[218] Arbelo E., Protonotarios A., Gimeno J.R., Arbustini E., Barriales-Villa R., Basso C., Bezzina C.R., Biagini E., Blom N.A., de Boer R.A. (2023). 2023 ESC Guidelines for the Management of Cardiomyopathies. Eur. Heart J..

[219] Shen L., Liu S., Zhang Z., Xiong Y., Lai Z., Hu F., Zheng L., Yao Y. (2024). Catheter Ablation of Ventricular Tachycardia in Patients with Arrhythmogenic Right Ventricular Cardiomyopathy and Biventricular Involvement. EP Eur..

[220] Corrado D., Leoni L., Link M.S., Della Bella P., Gaita F., Curnis A., Salerno J.U., Igidbashian D., Raviele A., Disertori M. (2003). Implantable Cardioverter-Defibrillator Therapy for Prevention of Sudden Death in Patients with Arrhythmogenic Right Ventricular Cardiomyopathy/Dysplasia. Circulation.

[221] Visser M., van der Heijden J.F., Doevendans P.A., Loh P., Wilde A.A., Hassink R.J. (2016). Idiopathic Ventricular Fibrillation. Circ. Arrhythmia Electrophysiol..

[222] Haïssaguerre M., Duchateau J., Dubois R., Hocini M., Cheniti G., Sacher F., Lavergne T., Probst V., Surget E., Vigmond E. (2020). Idiopathic Ventricular Fibrillation. JACC Clin. Electrophysiol..

[223] Wilde A.A.M., Garan H., Boyden P.A. (2019). Role of the Purkinje System in Heritable Arrhythmias. Heart Rhythm..

[224] Haïssaguerre M., Shoda M., Jaïs P., Nogami A., Shah D.C., Kautzner J., Arentz T., Kalushe D., Lamaison D., Griffith M. (2002). Mapping and Ablation of Idiopathic Ventricular Fibrillation. Circulation.

[225] Malhi N., Cheung C.C., Deif B., Roberts J.D., Gula L.J., Green M.S., Pang B., Sultan O., Konieczny K.M., Angaran P. (2019). Challenge and Impact of Quinidine Access in Sudden Death Syndromes: A National Experience. JACC Clin. Electrophysiol..

